# Context-Dependent Stability and Robustness of Genetic Toggle Switches with Leaky Promoters

**DOI:** 10.3390/life11111150

**Published:** 2021-10-28

**Authors:** Andras Gyorgy

**Affiliations:** Division of Engineering, New York University Abu Dhabi, Abu Dhabi P.O. Box 129188, United Arab Emirates; andras.gyorgy@nyu.edu; Tel.: +971-262-87348

**Keywords:** genetic switch, leaky promoter, multistability, robustness, context-dependence, scarce resources

## Abstract

Multistable switches are ubiquitous building blocks in both systems and synthetic biology. Given their central role, it is thus imperative to understand how their fundamental properties depend not only on the tunable biophysical properties of the switches themselves, but also on their genetic context. To this end, we reveal in this article how these factors shape the essential characteristics of toggle switches implemented using leaky promoters such as their stability and robustness to noise, both at single-cell and population levels. In particular, our results expose the roles that competition for scarce transcriptional and translational resources, promoter leakiness, and cell-to-cell heterogeneity collectively play. For instance, the interplay between protein expression from leaky promoters and the associated cost of relying on shared cellular resources can give rise to tristable dynamics even in the absence of positive feedback. Similarly, we demonstrate that while promoter leakiness always acts against multistability, resource competition can be leveraged to counteract this undesirable phenomenon. Underpinned by a mechanistic model, our results thus enable the context-aware rational design of multistable genetic switches that are directly translatable to experimental considerations, and can be further leveraged during the synthesis of large-scale genetic systems using computer-aided biodesign automation platforms.

## 1. Introduction

Living cells function as microscopic factories, converting energy and building blocks into a large array of products [1]. The vision of synthetic biology is to reliably control these processes by bringing together tools from multiple fields, ranging from biotechnology and genetic engineering to control/systems engineering and machine learning [2,3,4]. Recent breakthroughs highlight that this approach holds the promise of revolutionizing multiple sectors, with examples ranging from biocomputing [5] through biotherapeutics [6] to biofuel production [7].

While modifying the genetic blueprint of living organisms is now routine practice via genome editing, designing systems of even modest complexity still requires numerous iterative cycles and vast libraries [8,9] due to context-dependence [10,11], often leading to perplexing behavior [12,13]. For instance, as a result of metabolic burden, synthesizing heterologous proteins can lead to growth rate reduction and the expression of two unrelated proteins becoming coupled [14,15,16,17,18,19,20,21,22]. Moreover, due to host-circuit interaction, bistability [23] or even oscillations [24] may unexpectedly emerge. Tackling the issue of modularity thus requires system-level approaches [25] that combine a diverse set of quantitative tools [26,27,28,29,30,31,32,33,34,35,36].

**Figure 1:**
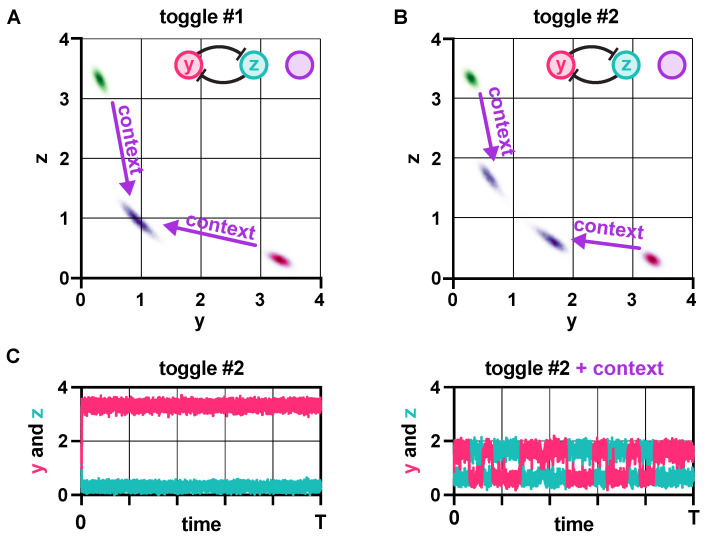
Stability and robustness of genetic switches depend on their context [40,41]. (**A**) In the absence of loading from its context, trajectories of toggle switch #1 converge to one of two metastable states (red and green). Once loading from the context is present, realized via the addition of the repressilator [42], the two distinct subpopulations coalesce (purple). (**B**) Although toggle switch #2 behaves identically to toggle switch #1 in the absence of the context (red and green), the same perturbation only causes a slight shift of the two subpopulations towards each other (purple). (**C**) Toggle switch #2 displays dramatically reduced robustness to noise due to its context. For more details on the stochastic simulations [43,44,45], see Section S1 and Section S6 in the SI.

Considering the central role that multistable switches play in both natural and synthetic gene systems [37,38,39], it is especially troubling that their behavior displays particularly strong dependence on their context [40,41]. While some variants fail to function as a memory module upon changes to their genetic context as a result of competition for shared resources (Figure 1A), others preserve this critical functionality (Figure 1B), although their robustness to noise decreases (Figure 1C). Therefore, core properties of the toggle switch are not only context-dependent but seemingly identical realizations can react fundamentally differently to burden-related perturbations (Figure 1).

Motivated by this, our objective here is two-fold: we seek to (i) characterize how tunable biophysical parameters of the toggle switch shape its stability and robustness properties, and to (ii) quantify how burden from the genetic context influences these fundamental characteristics. Significantly extending our prior results [46,47] by accounting for the general case of leaky promoters, the combined analytical/numerical approach presented here not only reveals surprising and previously unknown behaviors, but it also provides us with explicit design guidelines that promote modularity and increased robustness to noise. To illustrate the former, we uncover that while both promoter leakiness and resource sequestration separately act against bistability, their combined effect can surprisingly give rise to tristable dynamics even without positive feedback [48]. Regarding the latter, we demonstrate that while high resource sequestration inside the toggle switch pushes it towards monostability and diminished robustness to noise [46,47], it also decreases the burden-related effects of the context on the stability and robustness properties of the switch. Finally, as our results account for cell-to-cell variability ubiquitous to living systems [49], we reveal, for instance, that the correlation between protein expression and resource sequestration increases population-level uniformity.

To ensure predictable system-level behavior, the effects of context-dependence must be explicitly accounted for at the part level. To ensure that the stability and robustness of genetic toggle switches can be rationally adjusted by leveraging the explicit design guidelines we uncover, our approach relies on a mechanistic model [46,47] that accurately captures the limited availability of shared transcriptional and translational resources [16,50]. As a result, we can systematically engineer the performance of genetic switches by combining a wide variety of experimental tools such as RBS and promoter engineering [51,52], the introduction of decoy sites [53], and the expression of heterologous proteins [15,16].

This paper is organized as follows. After presenting the mathematical model and the technical tools underpinning our analysis, we reveal the role that promoter leakiness, scarcity of shared transcriptional and translational resources, and cell-to-cell heterogeneity collectively play to establish the fundamental stability and robustness properties of genetic switches. Following this, we illustrate how these results can be further generalized to account for loading from the genetic context of the switch.

## 2. Materials and Methods

### 2.1. Mathematical Model to Account for the Scarcity of Resources

The toggle switch represents one of the first rationally engineered genetic devices [54], comprising two proteins (*y* and *z*) repressing each other’s expression. Assuming that the repressors bind as dimers [32,38,55,56] and considering leaky promoters [52], the de-dimensionalized dynamics of the toggle switch are given by the following equations:

$$\dot{y}=\alpha \left(\nu +\frac{1}{1+z^{2}}\right)-y,\;\dot{z}=\alpha \left(\nu +\frac{1}{1+y^{2}}\right)-z,$$

where α and ν denote expression rate and promoter leakiness [57], respectively.

Although cellular resources (e.g., RNA polymerase and ribosomes) are shared among processes responsible for the production of *y* and *z*, the widely adopted model of the toggle switch above [54,57,58] fails to capture this major source of context-dependence [10]. Consequently, it is unable to explain the phenomena in Figure 1. To address this issue and capture the scarcity of shared resources [15,16,17,50], the dynamics of the toggle switch can be extended (SI Section S1) as follows:

(1) $$\begin{array}{cc} \dot{y}= & \frac{\alpha \left(\nu +\frac{1}{1+z^{2}}\right)}{1+\beta \left(2\nu +\frac{1}{1+y^{2}}+\frac{1}{1+z^{2}}\right)+\beta_{c}}-y=f_{y}\left(y,z\right),\\ \dot{z}= & \frac{\alpha \left(\nu +\frac{1}{1+y^{2}}\right)}{1+\beta \left(2\nu +\frac{1}{1+y^{2}}+\frac{1}{1+z^{2}}\right)+\beta_{c}}-z=f_{z}\left(y,z\right), \end{array}$$

where β characterizes resource usage required for the production of *y* and *z*; similarly, $\beta_{c}$ accounts for the burden originating from the context of the toggle switch, decreasing the effective production rate [13,16,50]. Therefore, the above model captures protein production, promoter leakiness, and resource competition both inside and outside the toggle switch via the lumped parameters α, ν, β, and $\beta_{c}$, respectively, motivated by in vivo and in vitro experimental data [13,15,16,17,50]. Importantly, these parameters can be easily tuned experimentally: e.g., α via RBS engineering [51], β via decoy sites [59], ν via promoter engineering [52], and $\beta_{c}$ via the expression of heterologous proteins [15,16]. While we consider a symmetric realization in this article (i.e., α, β, and ν are identical for *y* and *z*), this assumption can be relaxed to study the effects of parameter asymmetry [47,60].

Regarding the typical range of α, parameters of a single-copy toggle switch were tuned to ensure bistability in [52], which only happens when α>2 (SI Section S2.1). Since α is proportional to the plasmid copy number (SI Section S1), considering high copy number plasmids instead of the chromosomally integrated variant in [52], α can be increased substantially. Therefore, in this paper, we consider 0≤α≤300, which spans the typical range of plasmid copy number per cell [61]. As β decreases the effective production rate according to (1), based on experimental data presented in [15,16], we estimate that it typically lies within the range of 0≤β≤10 when expressing one or two genes. Importantly, β can be further increased by the addition of tandem and fused proteins [57] or decoy sites [59] by modifying the physical layout [12] or by activating a pathway downstream of the toggle switch comprising multiple genes, thus significantly reducing the effective production rate, an effect that is likely further amplified when relying on orthogonal resources available only in modest quantities [62]. To account for both simple and more complex circuits, we consider 0≤β≤40 in this paper, such that 0≤β≤10 corresponds to the former, whereas 10<β≤40 agrees with the latter.

Finally, it is important to note that promoter leakiness can be taken into account by considering a different formulation [63], which leads to the following changes in (1):

$$\alpha \left(\nu +\frac{1}{1+v^{2}}\right)\to \alpha^{\prime }\left(\nu^{\prime }+\frac{1-\nu^{\prime }}{1+v^{2}}\right),\;\beta \left(\nu +\frac{1}{1+v^{2}}\right)\to \beta^{\prime }\left(\nu^{\prime }+\frac{1-\nu^{\prime }}{1+v^{2}}\right).$$

Crucially, these two approaches lead to identical results considering $\alpha =\alpha^{\prime }\left(1-\nu^{\prime }\right)$, $\beta =\beta^{\prime }\left(1-\nu^{\prime }\right)$, and $\nu =\nu^{\prime }/\left(1-\nu^{\prime }\right)$. For more details on the chemical reactions and the model order reduction, see Section S1 in the SI.

### 2.2. Stability Analysis

Stability analysis of (1) when ν=0 has already been carried out [46,54]. The main technical steps underpinning the analysis are summarized in SI Sections S2.1 and S2.2. For instance, when ν=β=0, the corresponding dynamics in (1) have a single equilibrium when α<2, and three otherwise [54]. Moreover, in the case of the former, we conclude the following:

$$\frac{\partial f_{y}\left(y,z\right)}{\partial y}<0,\;\frac{\partial f_{y}\left(y,z\right)}{\partial z}<0,\;\frac{\partial f_{z}\left(y,z\right)}{\partial y}<0,\;\frac{\partial f_{z}\left(y,z\right)}{\partial z}<0,$$

together with

$${\left.\frac{dz}{dy}\right|}_{f_{y}\left(y,z\right)=0}<{\left.\frac{dz}{dy}\right|}_{f_{z}\left(y,z\right)=0}<0$$

at the unique equilibrium, so that invoking the implicit function theorem yields

$$\begin{array}{cc} 0> & \frac{\partial f_{y}\left(y,z\right)}{\partial y}+\frac{\partial f_{z}\left(y,z\right)}{\partial z},\\ 0< & \frac{\partial f_{y}\left(y,z\right)}{\partial y}\frac{\partial f_{z}\left(y,z\right)}{\partial z}-\frac{\partial f_{y}\left(y,z\right)}{\partial z}\frac{\partial f_{z}\left(y,z\right)}{\partial y}, \end{array}$$

thus, the trace and determinant of the Jacobian of (1) are negative and positive, respectively, certifying the stability of the unique fixed point [64]. Stability and instability in the latter case (α>2) can be concluded similarly (SI Section S2.1). Including the effects of competition for shared resources (β>0) modifies the above result via the following parameter:

(2) $$q=\frac{2(1+\beta )}{\alpha }.$$

In particular, the dynamics become bistable when q<1, and remains monostable otherwise [46] (SI Section S2.2).

To study the effects of promoter leakiness in Section S2.3 of the SI, we first consider β=0 and show analytically that the (α,ν) plane is partitioned into monostable and bistable regions using nullcline analysis, following the strategy presented above. Finally, in Section S2.4 of the SI we reveal that for ν>0.125 the dynamics become monostable, independent of the value of α and β, whereas for ν∈(0,0.125) numerical analysis reveals that the monostable, bistable, and tristable regions are separated by linear constraints of the form $\alpha a_{i}\left(\nu \right)-\beta +b_{i}\left(\nu \right)=0$, which can be written as $q_{i}\left(\nu \right)=1$ with

(3) $$q_{i}\left(\nu \right)=\frac{\beta -b_{i}\left(\nu \right)}{\alpha a_{i}\left(\nu \right)},\;i=1,2,3,$$

where $a_{i}\left(\nu \right)$ and $b_{i}\left(\nu \right)$ are ν-dependent parameters. For more details on the technical steps underpinning stability analysis, see Section S2 in the SI.

### 2.3. Robustness Analysis

If the system in (1) satisfied the constraint

(4) $$\frac{\partial f_{y}\left(y,z\right)}{\partial z}=\frac{\partial f_{z}\left(y,z\right)}{\partial y},$$

then we could define a potential V(y,z) such that

$$\frac{\partial V(y,z)}{\partial y}=-f_{y}\left(y,z\right),\;\frac{\partial V(y,z)}{\partial z}=-f_{z}\left(y,z\right).$$

Therefore, considering a sufficiently small time step ∆t, the changes in *y* and *z* would approximately be $∆y\approx \dot{y}∆t$ and $∆z\approx \dot{z}∆t$, yielding the following equation:

(5) $$∆V\left(y,z\right)=\frac{\partial V}{\partial y}∆y+\frac{\partial V}{\partial z}∆z=-\frac{dy}{dt}∆y-\frac{dz}{dt}∆z=-\left[f_{y}^{2}\left(y,z\right)+f_{z}^{2}\left(y,z\right)\right]∆t,$$

so that the potential surface V(y,z) could be computed along the trajectories of (1). Unfortunately, the condition (4) does not hold true for the dynamics in (1). Nonetheless, it was demonstrated in [65] that computing the quasi potential V(y,z) according to (5) still defines an epigenetic landscape on which trajectories flow “downhill” as ∆V(y,z)≤0 towards “valleys”, which corresponds to metastable fixed points where ∆V(y,z)=f(y,z)=f(z,y)=0. Therefore, the potential surface behaves similarly to a Lyapunov function [65,66].

With this, we can thus calculate the potential barriers separating metastable fixed points [47,67]. To this end, introduce x=(y,z) and let $x_{i}$ denote the metastable fixed points of (1) with the region of convergence $\Omega_{i}\in R^{2}$ (i.e., $x\left(t\right)\to x_{i}$ if $x\left(0\right)\in \Omega_{i}$ as t→∞). Then, the height of the potential barrier that trajectories need to overcome when crossing from $x_{i}$ to $x_{j}$ is $h_{i}=\inf_{\gamma }\sup_{x^{*}\in \gamma }V\left(x^{*}\right)-V\left(x_{i}\right)$, where γ denotes continuous paths leading from $x_{i}$ to $\Omega_{j}$. As the elevation on the potential landscape correlates with the likelihood of observing a given state, $h_{i}$ is inversely proportional to the mean transition time needed to cross from $x_{i}$ to $x_{j}$ [65]. Therefore, the frequency of transitions between metastable states can be characterized by approximating the underlying dynamics with a Markov jump process [68].

Stochastic simulations were performed by considering the overdamped Langevin dynamics widely used in biomolecular simulations [44,45], together with the Euler–Maruyama scheme [43]. For further details on the numerical algorithm used to compute the potential barriers and its dependence on model parameters, see Section S3 in the SI.

### 2.4. Population-Level Analysis

To model cellular heterogeneity, assume that (α,β)∼N(μ,Σ) with

$$\mu =\left(\begin{array}{c} \mu_{\alpha }\\ \mu_{\beta } \end{array}\right),\;\Sigma =\left[\begin{array}{cc} \sigma_{\alpha }^{2} & \rho \sigma_{\alpha }\sigma_{\beta }\\ \rho \sigma_{\alpha }\sigma_{\beta } & \sigma_{\beta }^{2} \end{array}\right],$$

where $\mu_{\alpha }$, $\mu_{\beta }$, $\sigma_{\alpha }$, $\sigma_{\beta }$ are the mean and standard deviation of α and β, respectively, and ρ is the correlation between them. Stability and robustness results from earlier still apply considering the following random variables:

(6) $$Q=\frac{2(1+\beta )}{\alpha },\;Q_{i}\left(\nu \right)=\frac{\beta -b_{i}\left(\nu \right)}{\alpha a_{i}\left(\nu \right)},\;i=1,2,3,$$

so that their particular realizations are given by *q* and $q_{i}\left(\nu \right)$ from (2) and (3).

With this, and relying on [69,70,71,72,73] in Sections S4.1 and S4.2 of the SI, we thus obtain that the cumulative distribution function of *Q* and $Q_{i}$ can be approximated as follows:

$$F\left(q\right)=P\left(Q<q\right)\approx \Phi \left(\frac{q-\mu_{Q}}{\sigma_{Q}\left(q\right)}\right),\;F_{i}\left(q_{i}\right)=P\left(Q_{i}<q_{i}\right)\approx \Phi \left(\frac{q_{i}-\mu_{i}}{\sigma_{i}\left(q_{i}\right)}\right),$$

where

(7) $$\begin{array}{ccc} \mu_{Q}= & \frac{2(1+\mu_{\beta })}{\mu_{\alpha }},\;\sigma_{Q}\left(q\right)= & \frac{\sqrt{q^{2}\sigma_{\alpha }^{2}+4\sigma_{\beta }^{2}-4q\rho \sigma_{\alpha }\sigma_{\beta }}}{\mu_{\alpha }},\\ \mu_{i}= & \frac{\mu_{\beta }-b_{i}\left(\nu \right)}{\mu_{\alpha }a_{i}\left(\nu \right)},\;\sigma_{i}\left(q_{i}\right)= & \frac{\sqrt{q^{2}{\left[a_{i}\left(\nu \right)\sigma_{\alpha }\right]}^{2}-2\rho q_{i}\left[a_{i}\left(\nu \right)\sigma_{\alpha }\right]\sigma_{\beta }+\sigma_{\beta }^{2}}}{a_{i}\left(\nu \right)\mu_{\alpha }}, \end{array}$$

and $a_{i}\left(\nu \right)$ and $b_{i}\left(\nu \right)$ are computed in Section S2.4 of the SI. With this, in Section S4.3 of the SI, we provide the analytic expressions of the probability of having monostable, bistable, and tristable dynamics: for instance, the probability of bistable dynamics is given by the following formula:

(8) $$p_{bi}=\Phi \left(\frac{1-\mu_{1}}{\sigma_{1}\left(1\right)}\right)-\Phi \left(\frac{1-\mu_{2}}{\sigma_{2}\left(1\right)}\right).$$

### 2.5. Context Effects

First, note that the rescalings $\alpha \leftarrow \alpha /(1+\beta_{c})$ and $\beta \leftarrow \beta /(1+\beta_{c})$ transform the dynamics in (1) as if the switch was isolated (i.e., as if $\beta_{c}$ was zero). In the absence of promoter leakiness (ν=0), the above rescalings increase the value of q=2(1+β)/α from (2); thus, the critical threshold of $\beta_{c}$ pushing the dynamics from bistability to monostability (q=1) also increases with β as $\beta_{c}^{\prime }=\left(1+\beta \right)\left(q^{-1}-1\right)$. Therefore, greater values of β protect against unwanted effects of loading from the context [47]. Similar results can also be obtained in the case of leaky promoters (ν>0) by considering the values of $q_{i}\left(\nu \right)$ from (3) to study how the stability and robustness properties of (1) change, and the results can be further extended to account for non-uniformity in $\beta_{c}$ by considering the case when α, β, and $\beta_{c}$ are drawn from a normal distribution, i.e., $\left(\alpha ,\beta ,\beta_{c}\right)∼N\left(\tilde{\mu },\tilde{\Sigma }\right)$. For more details, see Section S5 in the SI.

## 3. Results

In this section, we first reveal the role that each parameter plays in determining the stability properties of the toggle switch. Following this, we establish how biophysical parameters affect the robustness of metastable fixed points to noise, subsequently generalizing these results to account for parameter variations to study population-level effects. Finally, we uncover how additional burden from the genetic context of the toggle switch shapes the above relationships, explaining the phenomenon observed in Figure 1.

### 3.1. Stability Analysis

At its core, the toggle switch serves as a memory unit, provided that the underlying dynamics are multistable [54,74]. When competition for shared resources and leakiness are both neglected (β=ν=0), the toggle switch is bistable if α>2 [54] (SI Section S2.1), that is, sufficiently strong protein expression guarantees multistability. In what follows, we reveal how competition for shared resources and promoter leakiness shape this result (Section 2.2 in Materials and Methods).

**Figure 2:**
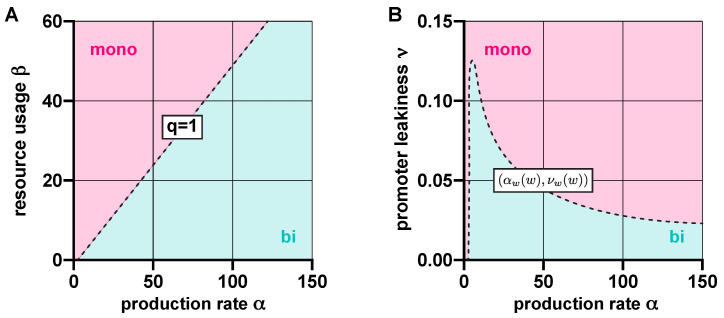
Resource competition and promoter leakiness both act against bistability. (**A**) In the absence of promoter leakiness, the toggle switch is bistable if q=2(1+β)/α<1, and monostable if otherwise (SI Section S2.2). (**B**) In the absence of competition for shared cellular resources, the dynamics are bistable if (α,ν) lies below the curve $\left(\alpha_{w}\left(w\right),\nu_{w}\left(w\right)\right)$, where $\alpha_{w}\left(w\right)={\left(1+w^{2}\right)}^{2}/\left(2w\right)$ and $\nu_{w}\left(w\right)=\left(w^{2}-1\right)/{\left(1+w^{2}\right)}^{2}$, parameterized by w≥1 (SI Section S2.3).

In the absence of leakiness (ν=0), the stability profile of (1) is determined by *q* from (2): the dynamics are bistable if q<1, and monostable if otherwise [46] (SI Section S2.2). That is, resource competition acts against bistability by increasing the value of *q* (Figure 2A). As illustrated in Figure 2B, leakiness plays a similar role when competition for shared resources is neglected (β=0) by promoting the emergence of monostability (SI Section S2.3).

**Figure 3:**
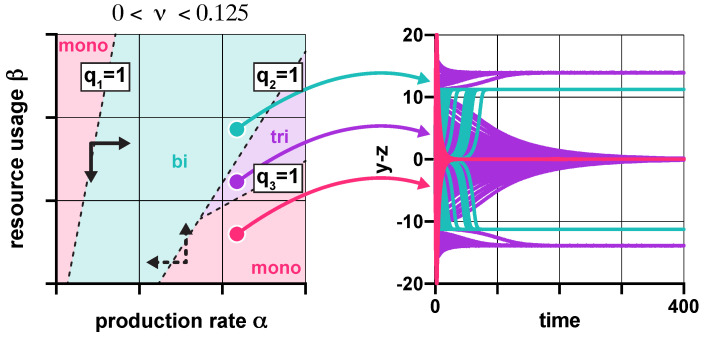
In the presence of promoter leakiness and resource competition, the constraints $q_{i}\left(\nu \right)=1$ from (3) partition the parameter space into monostable, bistable, and tristable regions (red, green, and purple, respectively). See SI Section S6 for simulation parameters.

Since resource competition and promoter leakiness both push the toggle switch towards monostability (Figure 2), it would be reasonable to expect that their synergistic effect simply results in a stronger thrust in the same direction. Surprisingly, this is not always true (SI Section S2.4): not only can these two factors counteract each other, but their compound effect can give rise to tristable dynamics, even in the absence of positive feedback [48]. While overwhelming leakiness (ν≥0.125) inevitably yields monostability, moderate levels (0<ν<0.125) can give rise to monostability, bistability, and even tristability. The corresponding regions in the (α,β) plane (Figure 3) are separated by the linear constraints $q_{i}\left(\nu \right)=1$, where $q_{i}\left(\nu \right)$ is defined in (3).

These results aid the rational design of multistable toggle switches by providing explicit guidelines to eliminate unwanted behaviors. For instance, decreasing promoter leakiness ν widens the green region in Figure 3 (SI Figure S4), thus facilitating the emergence of bistable dynamics, an effect already confirmed experimentally [52]. Similarly, while in the lower right monostable region in Figure 3, α and β must be decreased and increased, respectively (dashed arrows), in the top left monostable region in Figure 3, the parameters need to be tuned in the exact opposite direction to stimulate the emergence of bistability (solid arrows). Thus, our results reveal that understanding the source of unwanted monostability is of critical importance for the rational tuning of part-level biophysical parameters to ensure bistability.

### 3.2. Robustness Analysis

**Figure 4:**
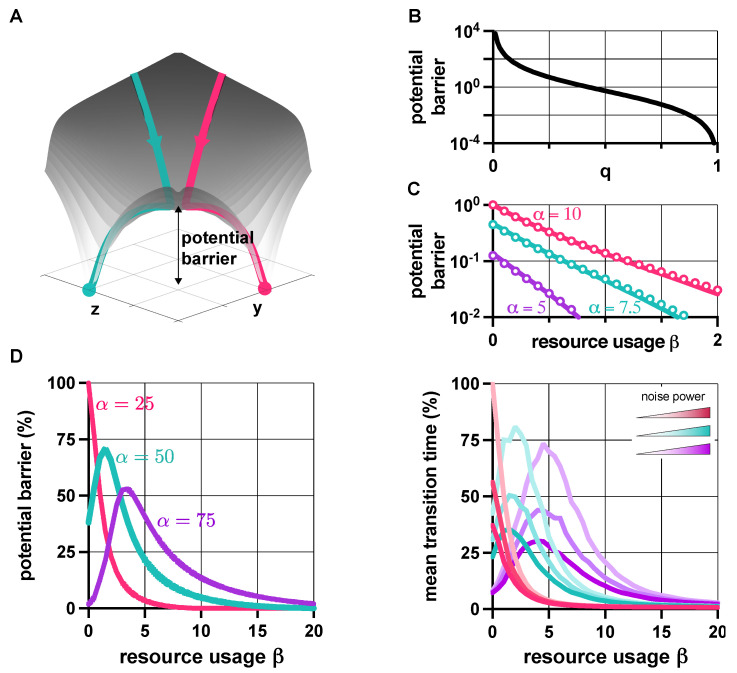
Robustness to noise is characterized using a potential landscape-based approach. (**A**) Trajectories flow downhill along the potential surface (grey) towards one of the metastable fixed points (red and green) depending on the initial conditions. (**B**) The potential barrier in the bistable case can be approximated by $h\approx \psi_{1}{\left(q^{-1}-1\right)}^{\psi_{2}}$ with $\left(\psi_{1},\psi_{2}\right)=\left(0.545,2.039\right)$, and 95% confidence intervals (0.544,0.547) and (2.035,2.042), respectively [67]. (**C**) This approximation (solid line) is well aligned with the data obtained from the numerical calculations of the potential barrier (circles). (**D**) The height of the potential barrier separating the two metastable fixed points in case of bistable dynamics for different values of α (normalized to the maximal value, represented as 100%), together with the mean transition time between these metastable fixed points (normalized to the maximal value, represented as 100%) in case of different noise power. See SI Section S6 for the simulation parameters.

We next reveal how protein production rate α, promoter leakiness ν, and β measuring resource sequestration together affect the potential barriers (Figure 4A) separating the metastable fixed points of (1) defined in Section 2.3 of Materials and Methods. As a result, we uncover how these factors influence the frequency of unwanted transitions (Figure 1C), and thus the reliability of the toggle switch in the presence of perturbations [65,66]. While in this study we focus on how noise causes trajectories to leave the metastable fixed points, it is important to note that noise can also trigger the onset of synchronization, for instance, by stabilizing a network of bistable systems to a common unstable equilibrium point [75,76].

We first focus on the role that α and β play, neglecting promoter leakiness (ν=0). Stochastic numerical simulations reveal that *q* from (2) not only determines the stability properties of the toggle switch (Figure 2A), but also its robustness to noise. In particular, the potential barrier separating the two metastable fixed points is well-approximated as $h\approx \psi_{1}{\left(q^{-1}-1\right)}^{\psi_{2}}$ with $\left(\psi_{1},\psi_{2}\right)=\left(0.545,2.039\right)$, as illustrated in Figure 4B. Therefore, as *q* decreases with α and increases with β, while greater production rate α yields reduced robustness to noise, increasing resource sequestration β has the opposite effect (Figure 4C).

To reveal the role of promoter leakiness, we first consider bistable dynamics (green in Figure 3A): as increasing ν acts against bistability (SI Figure S4), it is not surprising that it also yields decreased robustness to noise (SI Figure S5). Numerical analysis reveals (Figure S6 in the SI) that the optimal value of β maximizing robustness is a linear function of α, rotating counter-clockwise with ν. Thus, while β and ν both push the dynamics toward monostability (Figure 2), reduced robustness due to greater promoter leakiness can often be compensated by increasing β, as illustrated in the left panel of Figure 4D (Figure S7 in the SI), for instance, via the addition of decoy sites [53].

So far, we have focused on the potential barriers separating metastable fixed points relying on the quasi potential landscape calculated according to (5), as the height of these barriers determine their robustness to noise [65,66]. This is confirmed in the right panel of Figure 4D, depicting the average time that trajectories spend near a stable fixed point before transitioning to another due to noise (mean transition time). This result highlights the importance of studying not just the stability but also the robustness properties of genetic switches in order to avoid implementations with limited practical use due to frequent transitions between metastable fixed points.

### 3.3. Population-Level Analysis

As cellular heterogeneity is ubiquitous in living organisms [49], we next extend our results regarding the stability and robustness properties of (1) to account for cell-to-cell variability [73].

**Figure 5:**
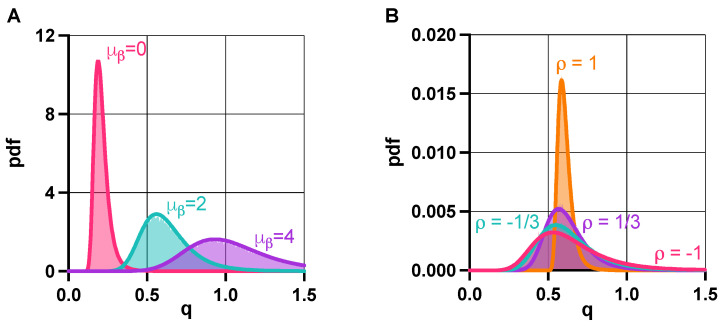
Population-level properties of the toggle switch are governed by the distribution of the random variable *Q* from (6) in the absence of promoter leakiness. (**A**) The distribution of *Q* shifts right as $\mu_{\beta }$ increases, pushing the population towards unimodality [73]. (**B**) Greater correlation ρ between α and β yields increased population-level uniformity. See SI Section S6 for the simulation parameters.

In the absence of promoter leakiness (ν=0), fundamental stability and the robustness properties of (1) are governed by *q* from (2), as illustrated in Figure 2A and Figure 4B. Accordingly, we study the distribution of *Q* as defined in (6), when the lumped parameters α and β are random variables to account for cell-to-cell variability (Section 2.4 in Materials and Methods). With this, the probability of bistability, or alternatively, the bistable fraction of the population is given by $p_{bi}=P\left(Q<1\right)$, revealing the role of tunable biophysical properties via (7) and (8). For instance, increasing the expected value $\mu_{\beta }$ of β pushes the distribution of *Q* to the right; thus, the population towards unimodality (Figure 5A), as expected from Figure 2A. What does not follow from previous results, as revealed by our analysis via (7) in Section 2.4 of Materials and Methods, is that the correlation ρ between α and β increases population-level uniformity, which is confirmed and illustrated in Figure 5B.

**Figure 6:**
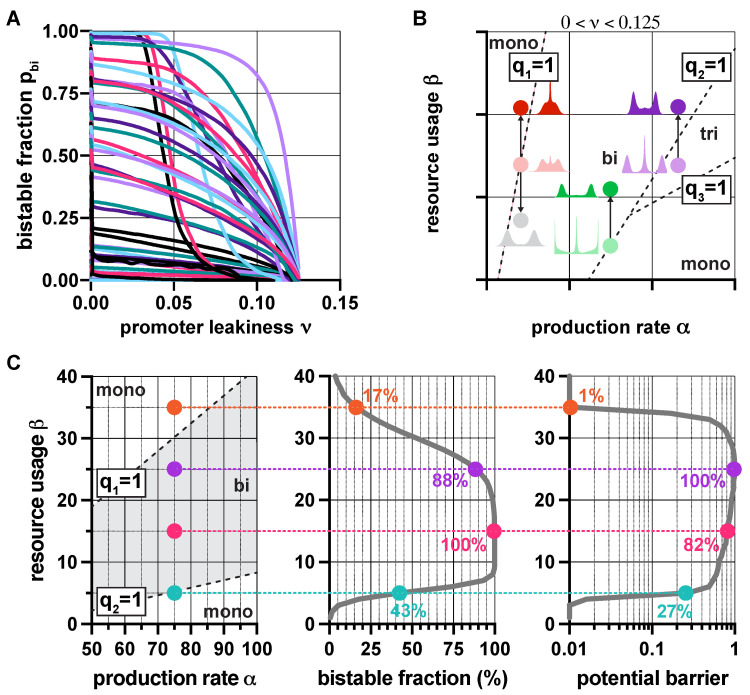
In the presence of promoter leakiness, population-level properties depend on the random variables $Q_{i}$ from (6). (**A**) Increasing leakiness decreases the bistable fraction of the population. (**B**) Unwanted trimodality can be eliminated, for instance, by increasing resource usage of the toggle switch for green/purple, and by decreasing it for red (Figure S9 in the SI). (**C**) Increasing the expected value $\mu_{\beta }$ of β first increases both the bistable fraction of the population (displayed in percentages) and the robustness of the metastable states to noise (measured via the potential barrier separating them), then this effect reverses as further increasing $\mu_{\beta }$ pushes the population away from the optimal region. See SI Section S6 for the simulation parameters.

Just as population-level characteristics follow from single-cell properties via *Q* when ν=0, a similar connection is established via the random variables $Q_{i}$ defined in (6) when relying on leaky promoters. In particular, the distribution of $Q_{i}$ can be approximated analytically (Section 2.4 in Materials and Methods) to reveal, for instance, that the bistable fraction $p_{bi}$ of the population decreases with promoter leakiness, as highlighted in Figure 6A.

Additionally, to illustrate how our results provide guidelines for eliminating unwanted behaviors, consider the population-level steady-state distributions of y−z in Figure 6B. Although those displayed in light colors all possess three peaks, the origin of this trimodality is not identical: while in the red/green cases it stems from mixing monostable and bistable subpopulations, in the purple case it is due to the underlying dominant tristable dynamics. Eliminating the unwanted middle peak (y≈z) thus requires different strategies. For instance, increasing the expected value $\mu_{\beta }$ of β (e.g., via decoy sites [53]) is the right choice in two cases (dark green and dark purple in Figure 6B), it is precisely the opposite of what is required in the third case, as this strategy yields an even more dominant middle peak (dark red in Figure 6B) instead of eliminating it, which can be achieved by decreasing $\mu_{\beta }$ (grey in Figure 6B). Robustness properties can be analyzed similarly, as illustrated in Figure 6C: for instance, while increasing $\mu_{\beta }$ first yields more pronounced bimodality and increased robustness to noise as we cross over the boundary $q_{2}\left(\nu \right)=1$ separating monostable and bistable dynamics, this trend quickly reverses when approaching the opposite transition (captured by $q_{1}\left(\nu \right)=1$).

### 3.4. Context Effects

**Figure 7:**
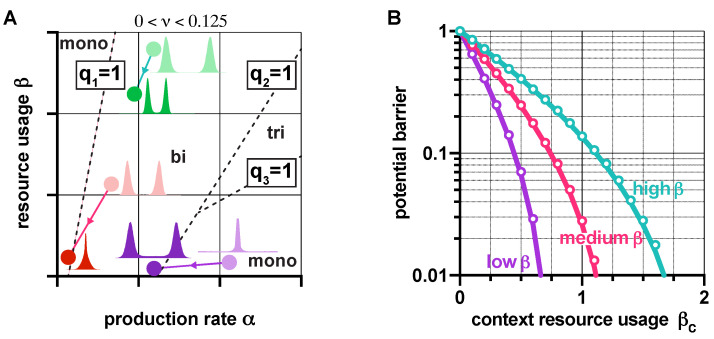
Loading from the context affects the stability and robustness properties of genetic switches. (**A**) Increasing $\beta_{c}$ causes a shift in the (α,β) plane towards the origin according to $\alpha \leftarrow \alpha /(1+\beta_{c})$ and $\beta \leftarrow \beta /(1+\beta_{c})$. Distributions show y−z in the steady state (for more details, see Figures S10 and S11 in the SI). (**B**) Higher β protects against loss of robustness to noise due to loading from the context (the parameters are chosen so that *q* is the same in all cases when $\beta_{c}=0$, yielding identical potential barriers). Solid lines correspond to predictions considering the approximation $h\approx \psi_{1}{\left(q^{-1}-1\right)}^{\psi_{2}}$ of the potential barrier with $\left(\psi_{1},\psi_{2}\right)=\left(0.545,2.039\right)$, whereas circles represent simulation data using the potential landscape directly. See SI Section S6 for the simulation parameters.

As the behavior of genetic switches displays strong dependence on their context [23,40,41], here we explore how loading from the context affects their stability and robustness properties. Since the rescalings $\alpha \leftarrow \alpha /(1+\beta_{c})$ and $\beta \leftarrow \beta /(1+\beta_{c})$ transform the dynamics in (1) as if the switch was isolated (i.e., as if $\beta_{c}$ was zero), revealing the effects of the context is straightforward, using results presented in earlier sections. For instance, in the absence of leakiness (ν=0), the value of *q* from (2) increases with $\beta_{c}$; hence, loading from the context pushes the dynamics towards monostability (Figure S9A in the SI). In the case of leaky promoters (ν>0), transitions between stability profiles happen when $q_{i}\left(\nu \right)=1$ is reached and crossed (Figure 7A). Thus, while bistability may be preserved in the presence of the context (shift from light to dark green in Figure 7A), additional loading could also trigger the loss of bistability (shift from light to dark red in Figure 7A).

Crucially, our results also expose that mitigating the unwanted effects of the context can be achieved by increasing β; for instance, increasing the critical threshold of $\beta_{c}$ (SI Section S5) that causes bistable toggle switches to behave as if they were monostable (at the expense of lowering robustness to noise if α is not increased simultaneously, see Figure 4). Similarly, high values of β lead to a smaller decrease in the potential barrier separating the metastable fixed points due to additional burden, and thus to a diminished increase of sensitivity to noise (Figure 7B). Naturally, these results directly inform us about the population-level behavior as well: bimodal populations can become unimodal when $\mu_{\beta }$ is low (red in Figure 7A), while those with high $\mu_{\beta }$ may remain bimodal when faced with an identical burden from their context (green in Figure 7A). More importantly, this phenomenon can also be leveraged, for instance, to render unwanted unimodal populations bimodal (purple in Figure 7A).

## 4. Discussion

Bioenergetic cost associated with the existence and expression of a gene is not a novel concept [77]. Recent advances in experimental techniques enable not only the precise characterization and subdivision of this burden [61,78,79], but also its accurate and multi-level control [80]. Complementing these high-throughput technologies, computational tools and quantitative models enable the design of complex biosystems [3,4,30,81].

To promote modularity and predictable system-level performance, sources of context-dependence need to be incorporated into the design of genetic modules [10]. Considering the prominent and versatile role that multistable genetic switches play in systems and synthetic biology [74], it is crucial to characterize how tunable biophysical parameters affect their stability and robustness properties, especially since they display particularly strong dependence on their context [23,40,41]. To address this issue, in this paper we considered a reduced order mechanistic model describing the dynamics of genetic switches, explicitly accounting for the limited availability of shared transcriptional/translational resources and yielding accurate predictions both in vivo [16] and in vitro [50], in addition to explaining counter-intuitive experimental phenomena [13].

Leveraging this, we not only revealed how tunable macroscopic parameters affect whether the toggle switch displays monostability, bistability, or tristability, but also how they shape the robustness of the metastable states to noise using a potential landscape-based approach [65]. In addition to confirming earlier experimental observations (e.g., minimizing promoter leakiness is crucial for bistability [52]), among the key findings of this paper are: (i) tristability can emerge as a result of the interplay between promoter leakiness and resource competition, even in the absence of positive feedback; (ii) while promoter leakiness always acts against bistability, resource sequestration could be leveraged to facilitate its emergence; and (iii) high internal resource sequestration can protect against burden arising from the genetic context.

In this paper, we thus derived explicit guidelines that aid the design of multistable genetic switches. Crucially, these results are directly translatable to experimental considerations due to the mechanistic model underpinning our results: e.g., α can be tuned via RBS engineering [51], β via the introduction of decoy sites [59], ν via promoter engineering [52], and $\beta_{c}$ via the expression of heterologous proteins [15,16]. Our findings complement recent efforts by mitigating the adverse effects of competition for shared cellular resources, for instance, by employing orthogonal resource pools [62,82,83], by relying on control theoretic strategies [84,85,86], and by splitting up multi-component genetic systems into smaller subcomponents distributed among multiple collaborative cell strains [87].

While competition for shared transcriptional/translational resources represents a major source of context-dependence [15,16], it is only one of many such sources. For instance, the limited availability of degradation machinery [88,89,90,91] and the spatial arrangement and orientation of compositional context [12] can result in crosstalk among otherwise unrelated genes. Similarly, metabolic burden can negatively impact cellular growth rate [15,18,84,92,93], although its extent depends on experimental conditions [94,95,96] and may only be transient as it often disappears after several generations of exponential growth [16,97]. Together with these phenomena and leveraging integrative circuit–host models [92,98,99], we expect our results to inform the rational design of individual switches relying on carefully characterized parts [63], as well as to be incorporated into the computer-aided fabrication of large-scale synthetic circuits [74,100].

## Data Availability

The MATLAB scripts required for obtaining the simulation data are available online at https://github.com/netbio-lab/leaky-toggle.git (accessed on 25 October 2021). All data were obtained using MATLAB R2021a, figures were prepared using Prism 9 and Adobe Illustrator 2021.

## Supplementary Materials

The following are available online at https://www.mdpi.com/article/10.3390/life11111150/s1, Text S1: Supporting Information detailing the underlying model, analysis, and simulation details.
