# Supplementary material for: Context-Dependent Stability and Robustness of Genetic Toggle Switches with Leaky Promoters
Source: Life (Basel). 2021 Oct 28;11(11):1150. doi: 10.3390/life11111150 (PMC8624834; doi:10.3390/life11111150)
Supplement: Supplementary file 1 [file life-11-01150-s001.zip › life-1424537-supplementary.pdf]

Context-dependent stability and robustness of genetic toggle switches with leaky promoters

Supporting Information

Andras Gyorgy*

Division of Engineering, New York University Abu Dhabi, United Arab Emirates

E-mail: andras.gyorgy@nyu.edu

Phone: +971 262 87348

This Supporting Information includes the technical details of the analysis underpinning the results presented in the manuscript. In particular, the following sections focus on (i) the mathematical model explicitly accounting for the limited availability of shared cellular resources; (ii) stability analysis; (iii) robustness analysis; (iv) effects of cell-to-cell heterogeneity; (v) extension of these results to account for context effects; (vi) simulation parameters, as detailed in the table of contents presented below.

Contents

S1 Mathematical model **2**

S1.1 Dynamics of protein expression with shared resources 2

S1.2 Dynamics of the toggle switch with scarce resources 5

S1.3 Toggle switch together with the repressilator 6

S2 Stability analysis **7**

S2.1 Resource competition and promoter leakiness are neglected 7

S2.2 Effects of resource competition 10

S2.3 Effects of promoter leakiness 10

S2.4 Effects of resource competition and promoter leakiness 13

S3 Robustness analysis **17**

S4 Population-level stability and robustness properties **21**

S4.1 Distribution of the ratio of normal random variables 21

S4.2 Approximate distribution of Q and Q_i 22

S4.3 Population-level composition 23

S5 Context effects **25**

S6 Simulation parameters **30**

References **32**

S1 Mathematical model

In this section, we introduce the mechanistic model underpinning our results, explicitly accounting for the limited availability of shared cellular resources [1], together with the time-scale separation based model order reduction leading to the dynamics of the toggle switch included in the manuscript. In particular, we first consider the expression of a single protein with a constitutive promoter, then generalize this setup to the case of multiple proteins with inducible promoters to derive the model of the toggle switch [2] in case of scarce resources. Finally, leveraging this model incorporating competition for shared resources, we illustrate the coupling between the toggle switch and its context represented by a repressilator module [3], leading to the results in Figure 1 of the manuscript.

S1.1 Dynamics of protein expression with shared resources

Consider first the expression of protein x from a constitutive promoter.

Transcription of mRNA m is modeled as

$$b + p \underset{\kappa_-}{\overset{\kappa^+}{\rightleftharpoons}} c, \qquad c \xrightarrow{\gamma^{TX}} b + p + m,$$

whereas translation of protein x is governed by the reactions

$$m + r \underset{k_-}{\overset{k^+}{\rightleftharpoons}} d, \qquad d \xrightarrow{\gamma^{TL}} m + r + x,$$

together with the decay/degradation of both mRNA and protein according to

$$m \xrightarrow{\delta^{TX}} \emptyset, \qquad d \xrightarrow{\delta^{TX}} \emptyset, \qquad x \xrightarrow{\delta^{TL}} \emptyset,$$

where the parameters and species are described in Table S1.

Therefore, the expression of protein x is governed by the dynamics

$$\begin{aligned} \dot{c} &= \left(\kappa^+ bp - \kappa^- c\right) - \gamma^{TX} c, \\ \dot{m} &= \gamma^{TX} c - \left(k^+ mr - k^- d\right) + \gamma^{TL} d - \delta^{TX} m, \\ \dot{d} &= \left(k^+ mr - k^- d\right) - \gamma^{TL} d - \delta^{TX} d, \\ \dot{x} &= \gamma^{TL} d - \delta^{TL} x, \end{aligned} \tag{S1}$$

together with the constraints

$$D = b + c, \qquad p_T = p + c, \qquad r_T = r + d, \tag{S2}$$

due to the conserved nature of DNA, RNAP and ribosome, where D, p_T, and r_T denote the total concentration of DNA, RNAP, and ribosomes, respectively.

Table S1: Microscopic biophysical parameters and species governing protein expression

Parameter	Description
x	protein concentration
p	concentration of free RNAP
r	concentration of free ribosome
b	promoter not bound with RNAP
c	promoter bound with RNAP
m	mRNA not bound with ribosome
d	mRNA bound with ribosome
γ^{TX}	transcriptional production rate constant
γ^{TL}	translational production rate constant
δ^{TX}	transcriptional decay rate constant
δ^{TL}	translational decay rate constant
κ^{+}	RNAP binding rate constant
κ^{-}	RNAP unbinding rate constant
k^{+}	ribosome binding rate constant
k^{-}	ribosome unbinding rate constant
D	total concentration of DNA
p_T	total concentration of RNAP
r_T	total concentration of ribosome

Next, introduce the dissociation constants

$$\kappa = \frac{\kappa^{-} + \gamma^{TX}}{\kappa^{+}}, \qquad k = \frac{k^{-} + \gamma^{TL} + \delta^{TX}}{k^{+}},$$

and note that binding and unbinding reactions are much faster than production/decay events, i.e., $\gamma^{TX} \ll \kappa^{-}$ and $\gamma^{TL}, \delta^{TX} \ll k^{-}$ [4], yielding $\kappa \approx \kappa^{-}/\kappa^{+}$ and $k = k^{-}/k^{+}$. Considering the quasi-steady state approximation of the dynamics in (S1) then yields

$$c = D\frac{\frac{p}{\kappa}}{1+\frac{p}{\kappa}}, \qquad d = \frac{mr}{k}, \qquad m = \frac{\gamma^{TX}}{\delta^{TX}}\frac{c}{1+\frac{r}{k}},$$

hence with the conservation laws for p_T and r_T from (S2) we obtain

$$p_T = p + D\frac{\frac{p}{\kappa}}{1+\frac{p}{\kappa}}, \qquad r_T = r + \frac{\gamma^{TX}}{\delta^{TX}}\frac{D\frac{\frac{p}{\kappa}}{1+\frac{p}{\kappa}}\frac{r}{k}}{1+\frac{r}{k}}.$$

Finally, since $p \ll \kappa$ and $r \ll k$ typically (see section B3 of [1] for more details) we have that

$$\dot{x} = \frac{\alpha}{1+\beta} - \delta^{TL} x \tag{S3}$$

with

$$\alpha = \frac{\gamma^{TX}\gamma^{TL} D p_T r_T}{\delta^{TX}\kappa k}, \qquad \beta = \frac{D}{\kappa}\left(1 + \frac{\gamma^{TX}}{\delta^{TX}}\frac{p_T}{k}\right). \tag{S4}$$

Therefore, considering the dynamics in (S3), the lumped parameter α captures the maximal expression rate of x, whereas its effective rate is given by $\alpha/(1+\beta)$. This effective rate increases, for instance, only sublinearly with the DNA copy number D, therefore, the denominator via the lumped parameter β captures the effect of resource sequestration associated with the expression of x (e.g., doubling D causes an increase of less than 100% in protein concentration at the steady state).

Similarly, in case of multiple proteins x_i for $i = 1, \ldots, N$ with inducible promoters, the expression in (S3) modifies according to

$$\dot{x}_i = \frac{\alpha_i \epsilon_i}{1 + \sum_{j=1}^{N} \beta_j \epsilon_j} - \delta^{TL} x_i, \tag{S5}$$

where α_j and β_j are defined as in (S4), and the activation levels ϵ_i can depend on $x_1, \ldots, x_N$ (for more details, see Section B in the SI of [5]).

S1.2 Dynamics of the toggle switch with scarce resources

In case of the toggle switch with proteins y and z and dissociation constants K_y and K_z to the promoter of z and y, respectively, the promoter activation levels are

$$\epsilon_y = \nu + \frac{1}{1 + \frac{z^2}{K_z^2}}, \qquad \epsilon_z = \nu + \frac{1}{1 + \frac{y^2}{K_y^2}},$$

where $\nu \geq 0$ represents the leakiness of the promoters. With this, from (S5) it follows that

$$\dot{y} = \frac{\alpha_y \epsilon_y}{1+\beta_y\epsilon_y+\beta_z\epsilon_z+\beta_c} - \delta^{TL} y, \qquad \dot{z} = \frac{\alpha_z \epsilon_z}{1+\beta_y\epsilon_y+\beta_z\epsilon_z+\beta_c} - \delta^{TL} z,$$

where $\beta_c = \sum_j \beta_j \epsilon_j$ denotes the burden due to the expression other than y and z (i.e., the genetic context of the toggle switch). Therefore, with the rescalings $y \leftarrow y/K_y$, $z \leftarrow z/K_z$, $\alpha_y \leftarrow \alpha_y/K_y$, $\alpha_z \leftarrow \alpha_z/K_z$, and $t \leftarrow \delta^{TL} t$ we obtain the de-dimensionalized dynamics

$$\begin{aligned} \dot{y} =& \frac{\alpha_y \left(\nu + \frac{1}{1+z^2}\right)}{1+\beta_y\left(\nu+\frac{1}{1+z^2}\right)+\beta_z\left(\nu+\frac{1}{1+y^2}\right)+\beta_c} - y, \\ \dot{z} =& \frac{\alpha_z \left(\nu + \frac{1}{1+y^2}\right)}{1+\beta_y\left(\nu+\frac{1}{1+z^2}\right)+\beta_z\left(\nu+\frac{1}{1+y^2}\right)+\beta_c} - z. \end{aligned} \tag{S6}$$

In the manuscript, we focus on the symmetric realization of the toggle switch: i.e., $\alpha = \alpha_y = \alpha_z$ and $\beta = \beta_y = \beta_z$, but a similar approach can be followed to study the effects of parameter asymmetry [6, 7]. In Sections S2–S4 we assume that $\beta_c = 0$ as effects of loading from the context are considered in Section S5.

S1.3 Toggle switch together with the repressilator

In case of the repressilator [3] with proteins x_i for $i = 1, 2, 3$ and corresponding dissociation constant K_i to the respective promoters, the activation levels are

$$\epsilon_1 = \frac{1}{1+\frac{x_3^2}{K_3^2}}, \qquad \epsilon_2 = \frac{1}{1+\frac{x_1^2}{K_1^2}}, \qquad \epsilon_3 = \frac{1}{1+\frac{x_2^2}{K_2^2}}.$$

Therefore, with the lumped parameters α_i and β_i defined in (S4), and with the rescalings $x_i \leftarrow x_i/K_i$, $\alpha_i \leftarrow \alpha_i/K_i$, and $t \leftarrow \delta^{TL} t$ from (S5) it follows that the dynamics of the

repressilator are given by

$$\begin{aligned}
\dot{x}_1 =& \frac{\frac{\alpha_1}{1+x_3^n}}{1+\frac{\beta_2}{1+x_1^n}+\frac{\beta_3}{1+x_2^n}+\frac{\beta_1}{1+x_3^n}+\beta_c'} - x_1,\\
\dot{x}_2 =& \frac{\frac{\alpha_2}{1+x_1^n}}{1+\frac{\beta_2}{1+x_1^n}+\frac{\beta_3}{1+x_2^n}+\frac{\beta_1}{1+x_3^n}+\beta_c'} - x_2,\\
\dot{x}_3 =& \frac{\frac{\alpha_3}{1+x_2^n}}{1+\frac{\beta_2}{1+x_1^n}+\frac{\beta_3}{1+x_2^n}+\frac{\beta_1}{1+x_3^n}+\beta_c'} - x_3,
\end{aligned} \tag{S7}$$

where β_c' denotes the loading due to the expression other than x_i for $i = 1, 2, 3$. In particular, when the toggle switch and the repressilator proteins are co-expressed, representing each other's context, we have that

$$\beta_c = \frac{\beta_2}{1+x_1^n}+\frac{\beta_3}{1+x_2^n}+\frac{\beta_1}{1+x_3^n}, \qquad \beta_c' = \beta\left(2\nu+\frac{1}{1+z^2}+\frac{1}{1+y^2}\right),$$

thus coupling together (S6) and (S7) due to the scarcity of shared cellular resources, leading to the data presented in Figure 1 of the manuscript.

S2 Stability analysis

Here we provide the details of the stability analysis of the toggle switch accounting for both resource competition and promoter leakiness via β and ν, respectively. We first focus on the case when both these factors are neglected, followed by the analysis when their effects are analyzed separately, and finally, we consider their combined impact.

S2.1 Resource competition and promoter leakiness are neglected

When resource competition and promoter leakiness are both neglected ($\beta = \nu = 0$), the dynamics of the toggle switch simplify to

$$\dot{y} = f_0(y,z), \qquad \dot{z} = f_0(z,y), \qquad f_0(v,w) = \frac{\alpha}{1+w^2} - v, \tag{S8}$$

yielding bistability when $\alpha > 2$ and monostability otherwise [2]. To facilitate the comprehension of later results, here we briefly summarize the main steps to achieve this result.

Number of fixed points From (S8) we obtain that $\alpha = z(1+y^2) = y(1+z^2)$, hence we have that $z^2 - z\,(y + 1/y) + 1 = 0$, leading to the constraint

$$z = \frac{\left(y + \dfrac{1}{y}\right) \pm \sqrt{\left(y + \dfrac{1}{y}\right)^2 - 4}}{2} = \frac{\left(y + \dfrac{1}{y}\right) \pm \left(y - \dfrac{1}{y}\right)}{2}.$$

Given that it is not possible to have $y = 0$ or $z = 0$ at an equilibrium of (S8), we obtain that we must have either $z = y$ or $z = 1/y$ at fixed points of (S8). In the former case when $z = y$, we obtain from (S8) that $z^3 + z - \alpha = 0$ with a negative discriminant, yielding one real positive solution and a pair of complex conjugates. Conversely, in the latter case when $z = 1/y$, from (S8) it follows that we must satisfy $z^3 - \alpha z^2 + z = 0$ at an equilibrium. Since $z \neq 0$, this leads to $z^2 - \alpha z + 1 = 0$ with its solutions given by $z = (\alpha \pm \sqrt{\alpha^2 - 4})/2$, thus (S8) has one positive fixed point if $\alpha < 2$ and three if $\alpha > 2$, as illustrated in Figure S1.

Stability of fixed points Stability can be determined using nullcline analysis, for instance, as detailed in Section 5.3 of [4]. In particular, from (S8) it follows that

$$\frac{\partial f_0(y,z)}{\partial y} < 0, \qquad \frac{\partial f_0(y,z)}{\partial z} < 0, \qquad \frac{\partial f_0(z,y)}{\partial y} < 0, \qquad \frac{\partial f_0(z,y)}{\partial z} < 0,$$

and at the equilibria denoted by circles in Figure S1 we have

$$\left.\frac{\mathrm{d}z}{\mathrm{d}y}\right|_{f_0(y,z)=0} < \left.\frac{\mathrm{d}z}{\mathrm{d}y}\right|_{f_0(z,y)=0} < 0,$$

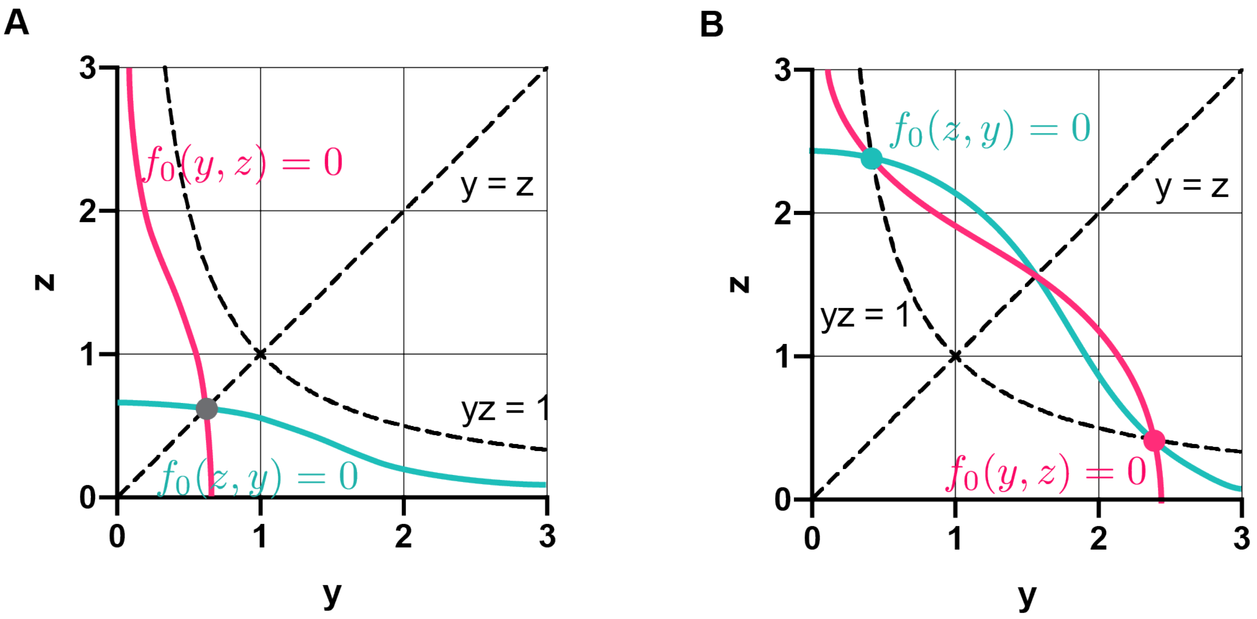

Figure S1: The stability profile of (S8) depends on the value of α. (A) The dynamics are monostable when $\alpha < 2$ as the nullclines $0 = f_0(y, z)$ and $0 = f_0(z, y)$ intersect only once (grey circle). (B) Once $\alpha > 2$, the nullclines intersect three times, rendering the dynamics bistable as the intersections denoted by red and green circles give rise to stable fixed points.

Combining these with the implicit function theorem yields that

$$\begin{aligned}0 &> \frac{\partial f_0(y,z)}{\partial y} + \frac{\partial f_0(z,y)}{\partial z},\\ 0 &< \frac{\partial f_0(y,z)}{\partial y}\frac{\partial f_0(z,y)}{\partial z} - \frac{\partial f_0(y,z)}{\partial z}\frac{\partial f_0(z,y)}{\partial y},\end{aligned}$$

that is, the trace and determinant of the Jacobian of (S8) are negative and positive, respectively, at the fixed points denoted by circles in Figure S1. Hence, the corresponding equilibria are stable and hyperbolic. Instability of the third fixed point in Figure S1B can be shown similarly.

S2.2 Effects of resource competition

Once resource competition is accounted for ($\beta > 0$), but promoter leakiness is still neglected ($\nu = 0$), the dynamics of the toggle switch from (S6) become

$$\dot{y} = \frac{\frac{\alpha}{1+z^2}}{1 + \frac{\beta}{1+z^2} + \frac{\beta}{1+z^2}} - y, \qquad \dot{z} = \frac{\frac{\alpha}{1+y^2}}{1 + \frac{\beta}{1+z^2} + \frac{\beta}{1+z^2}} - z. \tag{S9}$$

As it was originally shown in [8], the above dynamics are bistable if $q < 1$ with

$$q = \frac{2(1+\beta)}{\alpha},$$

and monostable otherwise.

S2.3 Effects of promoter leakiness

When promoter leakiness is taken into account ($\nu > 0$) but resource competition is neglected ($\beta = 0$), from (S6) the dynamics are given by

$$\dot{y} = \alpha\left(\nu + \frac{1}{1+z^2}\right) - y, \qquad \dot{z} = \alpha\left(\nu + \frac{1}{1+y^2}\right) - z, \tag{S10}$$

which is bistable if (α, ν) lies below the curve $(\alpha_w(w), \nu_w(w)$ in the (α, ν)-plane where

$$\alpha_w(w) = \frac{\left(1+w^2\right)^2}{2w}, \qquad \nu_w(w) = \frac{w^2 - 1}{\left(1+w^2\right)^2}, \qquad w \geq 1. \tag{S11}$$

To show this, note that $y = 0$ or $z = 0$ is not possible at an equilibrium of (S10), thus we must have $y(\nu + \frac{1}{1+y^2}) = z(\nu + \frac{1}{1+z^2})$, yielding

$$(y - z)\left(\nu y^2 z^2 + \nu y^2 + \nu z^2 - yz + \nu + 1\right) = 0.$$

Furthermore, at an equilibrium of (S10) we also have $\alpha\nu = y - \frac{\alpha}{1+z^2} = z - \frac{\alpha}{1+y^2}$, yielding

$$(y-z)\left[\left(1+y^2\right)\left(1+z^2\right) - \alpha\left(y+z\right)\right] = 0. \tag{S12}$$

Therefore, at an equilibrium of (S10) we either have $y = z$ or (y, z) must satisfy

$$0 = \left(1+y^2\right)\left(1+z^2\right) - \alpha\left(y+z\right), \tag{S13}$$

$$0 = \nu y^2 z^2 + \nu y^2 + \nu z^2 - yz + \nu + 1. \tag{S14}$$

In the former case when $y = z$, from (S10) it follows that $y = \alpha(\nu + \frac{1}{1+y^2})$, where the right-hand side and left-hand side are monotonically increasing and decreasing, respectively. Combining this with the fact that the right-hand side exceeds the left-hand side when $y = 0$, we conclude that (S10) has a single positive fixed point when $y = z$.

In the latter case, the pair (y, z) must satisfy both (S13) and (S14). As the former only depends on α, whereas the latter is only affected by ν, the effects of α and ν can be studied independently (Figure S2). Focusing first on the constraint (S13), it has two intersections with the $y = z$ line. Let (w', w') denote the one farther from the origin (red dot in Figure S2A). Next, considering the constraint (S14), define its intersections with the $y = z$ line as $w_- < w_+$, depicted in green and purple in Figure S2B, respectively, given by $w_{+,-} = \sqrt{1 - 2\nu \pm \sqrt{1-8\nu}}/(2\nu)$. Therefore, $w_- \to 1$ and $w_+ \to \infty$ for $\nu \to 0$; at $\nu = 0.125$ we obtain that $w_- = w_+ = \sqrt{3}$; and for greater values of ν the points w_- and w_+ become imaginary. Furthermore, from (S14) it follows that for a given value of w, the corresponding value of α is given by $\alpha_w(w)$ from (S11), depicted in Figure S2C. Similarly, from (S13) we obtain that when $y = z = w$, the corresponding value of ν is given by $\nu_w(w)$ from (S11), depicted in Figure S2D. Therefore, considering the curves in Figure S2A and Figure S2B, for a particular (α, ν) pair the curves intersect exactly twice if $w_- < w < w_+$. Thus, for a given α, we can determine the corresponding value of w' (Figure S2C), hence the value of ν according to (S11). This value defines the critical threshold for leakiness, as exceeding it

causes the condition $w_- < w < w_+$ to be violated (Figure S2D), eliminating the existence of solutions satisfying (S13)–(S14).

Summarizing the above,: the constraints (S13) and (S14) intersect exactly twice if and only if (α, ν) lies below the curve $(\alpha_w(w), \nu_w(w))$ defined in (S11). Combining this with the fact that (S10) has exactly one fixed point such that $y = z$, we conclude that the above

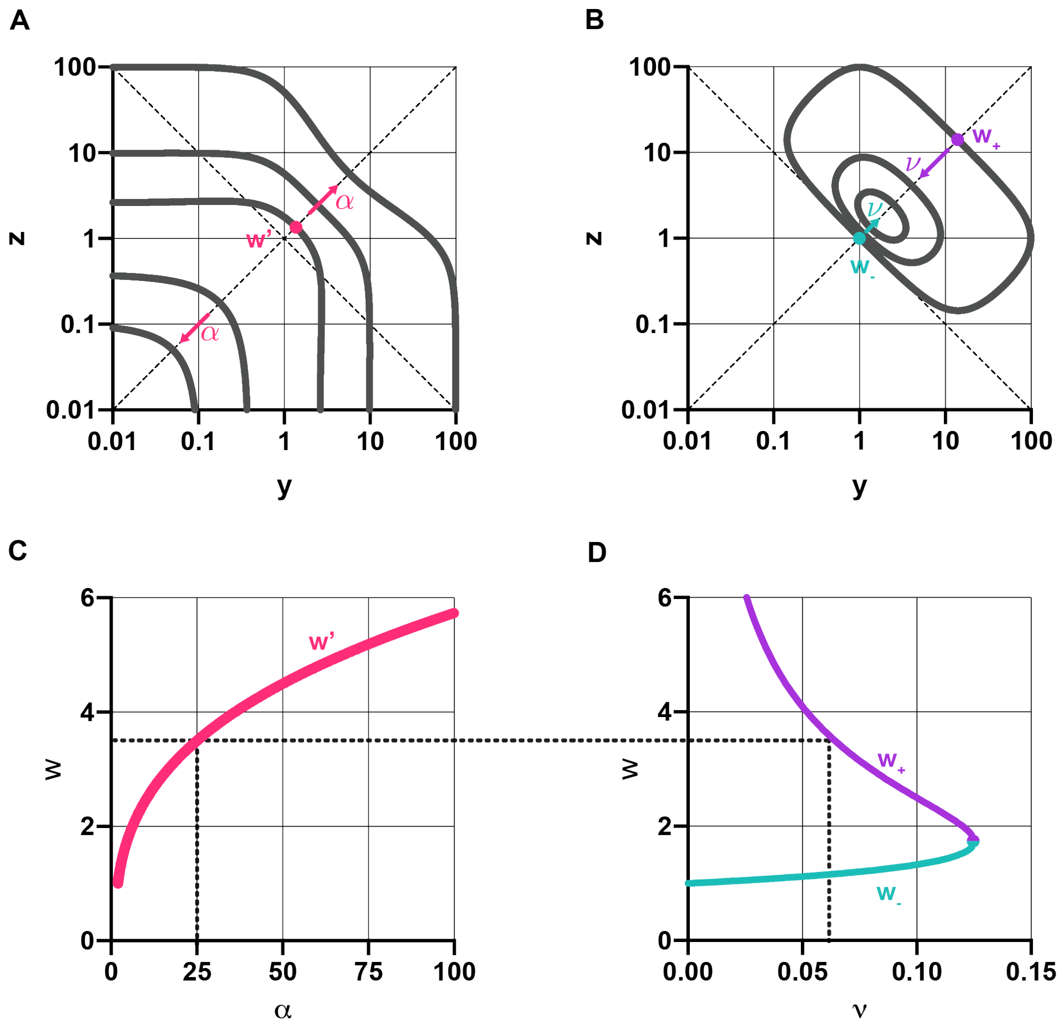

Figure S2: Promoter leakiness shapes the stability profile of the toggle switch via the constraints (S13) and (S14). (A) Pairs of (y, z) satisfying (S14) for different values of α ($\alpha = 3$, $\alpha = 10$, and $\alpha = 100$, respectively, the red arrow denotes the direction in which α increases). (B) Pairs of (y, z) satisfying (S13) for different values of ν ($\nu = 0.005$, $\nu = 0.05$, and $\nu = 0.1$, respectively, the green and purple arrows denote the direction in which ν increases). (C) Intersection of (S13) with the $y = z$ line, denoted by the red dot in panel A. (D) Intersection of (S14) with the $y = z$ line, denoted by the green/purple dots in panel B.

condition also guarantees the existence of three fixed points, otherwise there is only one. Stability of the fixed points can be concluded as in the previous section, eventually leading to the following: (S10) is bistable if (α, ν) lies below the curve $(\alpha_w(w), \nu_w(w))$ defined in (S11), otherwise it is monostable, as summarized in Figure 2B of the manuscript.

S2.4 Effects of resource competition and promoter leakiness

Once both resource competition and promoter leakiness are considered ($\beta, \nu > 0$), the dynamics of the toggle switch become

$$\dot{y} = \frac{\alpha\left(\nu + \frac{1}{1+z^2}\right)}{1+\beta\left(2\nu + \frac{1}{1+z^2} + \frac{1}{1+y^2}\right)} - y, \qquad \dot{z} = \frac{\alpha\left(\nu + \frac{1}{1+y^2}\right)}{1+\beta\left(2\nu + \frac{1}{1+z^2} + \frac{1}{1+y^2}\right)} - z. \tag{S15}$$

As it is not possible to have $y = 0$ or $z = 0$ at an equilibrium of (S15), in what follows we assume that $y, z > 0$. To establish the stability profile of (S15), first note that at an equilibrium we have

$$\begin{aligned} \alpha\nu =& y\left[1+\beta\left(2\nu + \frac{1}{1+z^2} + \frac{1}{1+y^2}\right)\right] - \frac{\alpha}{1+z^2}, \\ \alpha\nu =& z\left[1+\beta\left(2\nu + \frac{1}{1+z^2} + \frac{1}{1+y^2}\right)\right] - \frac{\alpha}{1+y^2}, \end{aligned}$$

yielding

$$(y-z)\left[1+\beta\left(2\nu + \frac{1}{1+z^2} + \frac{1}{1+y^2}\right)\right] - \alpha\left(\frac{1}{1+z^2} - \frac{1}{1+y^2}\right) = 0,$$

which can be rewritten as

$$(y-z)\left[(1+2\beta\nu)\left(1+y^2\right)\left(1+z^2\right) + \beta\left(2+y^2+z^2\right) - \alpha(y+z)\right] = 0. \tag{S16}$$

Second, from (S15) it also follows that at an equilibrium we have

$$\frac{\alpha}{1+\beta\left(2\nu+\frac{1}{1+z^2}+\frac{1}{1+y^2}\right)}=\frac{y}{\nu+\frac{1}{1+z^2}}, \qquad \frac{\alpha}{1+\beta\left(2\nu+\frac{1}{1+z^2}+\frac{1}{1+y^2}\right)}=\frac{z}{\nu+\frac{1}{1+y^2}},$$

yielding $y(\nu+\frac{1}{1+y^2})=z(\nu+\frac{1}{1+z^2})$, which can be rewritten as

$$(y-z)\left(\nu y^2z^2+\nu y^2+\nu z^2-yz+\nu+1\right)=0. \tag{S17}$$

Considering (S16)–(S17), there are two cases: when $y=z>0$ and when $y\neq z$. In the former case, at a fixed point of (S15) we have that

$$y=\frac{\alpha\left(\nu+\frac{1}{1+y^2}\right)}{1+2\beta\left(\nu+\frac{1}{1+y^2}\right)}, \tag{S18}$$

where the left-hand side is monotonically increasing with y, and conversely, the right-hand side is decreasing. Since the latter exceeds the former when $y=0$, we conclude that (S18) has a single positive root, hence (S15) has a unique positive fixed point when $y=z$.

Next, we focus on the case when $y\neq z$. Since $1+2\beta\nu>0$, it follows from (S16)–(S17) that fixed points of (S15) must satisfy

$$0=\nu y^2z^2+\nu y^2+\nu z^2-yz+\nu+1, \tag{S19}$$

$$0=\left(1+y^2\right)\left(1+z^2\right)+\tilde{\beta}\left(2+y^2+z^2\right)-\tilde{\alpha}\left(y+z\right), \tag{S20}$$

with $\tilde{\alpha}=\alpha/(1+2\beta\nu)$ and $\tilde{\beta}=\beta/(1+2\beta\nu)$. Therefore, the stability profile of (S15) is determined by the number of intersections that (S19)–(S20) have. The case of $\nu=0$ has been studied in Section S2.2, and since (S19) has no real solutions for $\nu>0.125$, here we focus on the case when $0<\nu<0.125$. Within this range, numerical analysis reveals that (S19)–(S20) have either zero, two, or four intersections (apart from degenerate cases). Nullcline analysis similar to the one presented in Section S2.1 further uncovers that these

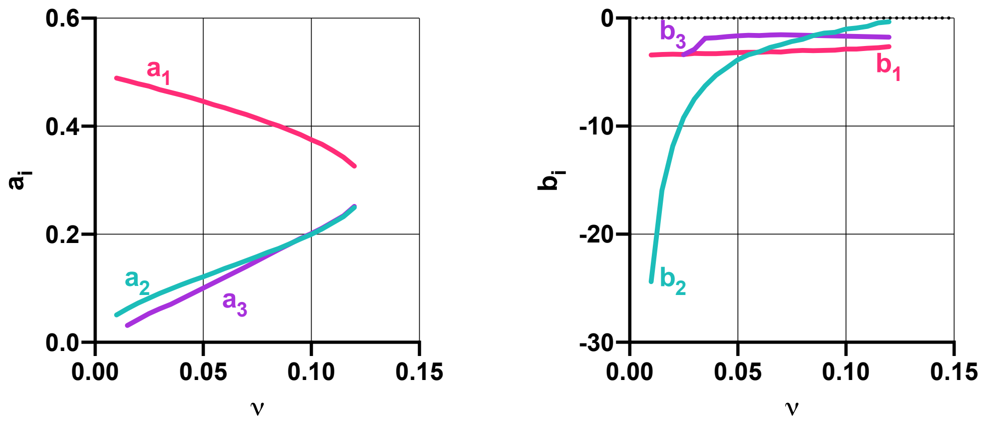

Figure S3: The stability profiles are computed numerically for 1,000,000 samples from the range $(\alpha, \beta) \in [0, 1000] \times [0, 100]$ for all values of ν. In all cases, the Pearson correlation coefficient is $R^2 > 0.99$ for the linear fit $\alpha a_i(\nu) - \beta + b_i(\nu) = 0$ to estimate $a_i(\nu)$ and $b_i(\nu)$.

correspond to monostable, bistable, and tristable dynamics, respectively. Importantly, the regions in which (S19)–(S20) have zero, two, or four intersections are separated by linear constraints given by $q_i(\nu) = 1$ with

$$q_i(\nu) = \frac{\beta - b_i(\nu)}{\alpha a_i(\nu)} \qquad i = 1, 2, 3,$$

where $a_i(\nu)$ and $b_i(\nu)$ depend on ν, as detailed in Figure S3. In particular, we have that (S15) is (i) monostable if $q_1 > 1$ or $\max(q_2, q_3) < 1$; (ii) bistable if $q_1 < 1 < q_2$; and (iii) tristable if $q_2 < 1 < q_3$, as illustrated in Figure 3 in the main text. Therefore, by studying how the constraints are affected by ν (Figure S4), we can reveal how the interplay among α, β, and ν shape the stability profile of (S15).

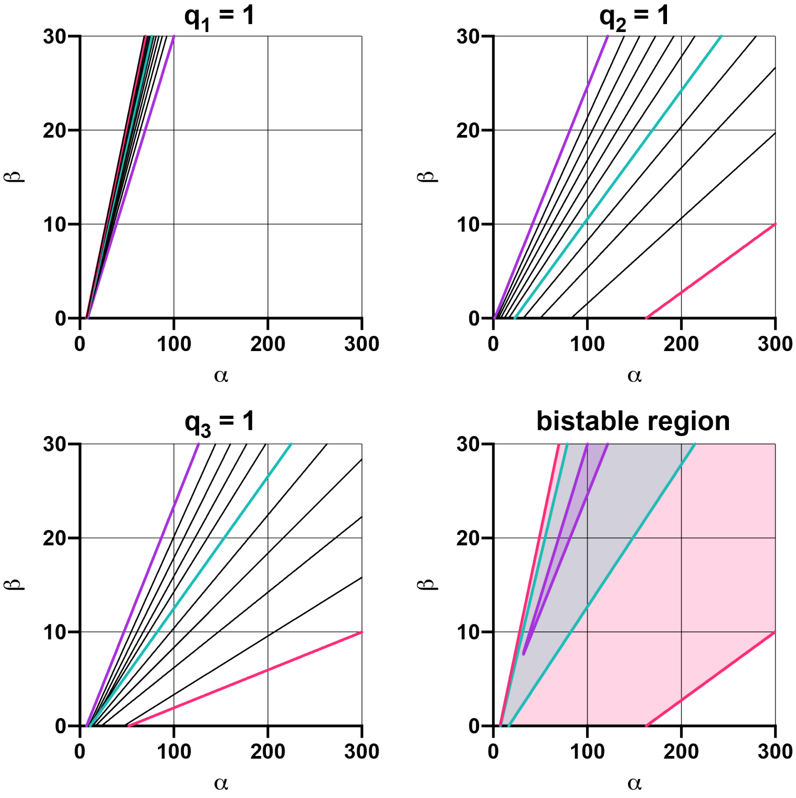

Figure S4: The constraint $q_1(\nu) = 1$ rotates clockwise with ν, while $q_2(\nu) = 1$ and $q_3(\nu) = 1$ both rotate counter-clockwise. Thus, the bistable region between $q_1(\nu) = 1$ and $q_2(\nu) = 1$ shrinks as ν increases. Red, green, and purple correspond to $\nu = 0.02$, $\nu = 0.07$, and $\nu = 0.12$, respectively.

S3 Robustness analysis

Having uncovered how the stability profile of (S15) is shaped by the interplay among α, β, and ν, here we characterize their effect on the robustness of the metastable fixed points to noise. To this end, we first calculate the potential barrier separating them as it is inversely proportional to the average time it takes for trajectories to cross from one to another [9]. According to the definition of $V(y, z)$ and h_i in Section 2.3 of the manuscript, for each metastable fixed point [9, 10]:

1. determine the number and location of fixed points, and classify them as stable/unstable (as detailed in Section S2);

2. create a set $\mathcal{S}$ of initial conditions in the range $(y, z) \in [\alpha, \alpha]$ (e.g., uniform 2D grid), simulate the system trajectories for the initial points in $\mathcal{S}$, compute the potential decrease along them according to its definition in Section 2.3 of the manuscript, and partition the endpoints of these trajectories into as many clusters as the number of stable fixed points (e.g., using k-means clustering in MATLAB);

3. for each region of convergence, determine the potential of the initial points by assigning the same potential to the endpoints (stable fixed point), then adjust the potentials in each region of convergence so that trajectories starting nearby (at the edges of the regions of convergence) have the same potential;

4. calculate the potential barriers according the definition of h_i in Section 2.3 of the manuscript.

With this, we next present numerical simulation results regarding the potential barriers that separate the metastable fixed points of (S15). To this end, let x_1 and x_2 denote the metastable fixed points in the bistable case, whereas x_3 corresponds to the third equilibrium appearing once the dynamics become tristable, so that $h_1 = h_2$ and h_3 denote the potential barriers required for leaving the former two and the latter, respectively. In Figure S5 we

illustrate how the potential barriers $h_1 = h_2$ depend on α and β in the region of bistability (i.e., when $q_1(\nu) < 1$ and $q_2(\nu) > 1$, see Section S2.4) for different values of ν in the range of multistability (i.e., $0 < \nu < 0.125$). Importantly, the optimal value β^* of β maximizing the potential barriers $h_1 = h_2$ increases linearly with α for a given value of ν and this constraint rotates counter-clockwise with increasing ν, as illustrated in Figure S6.

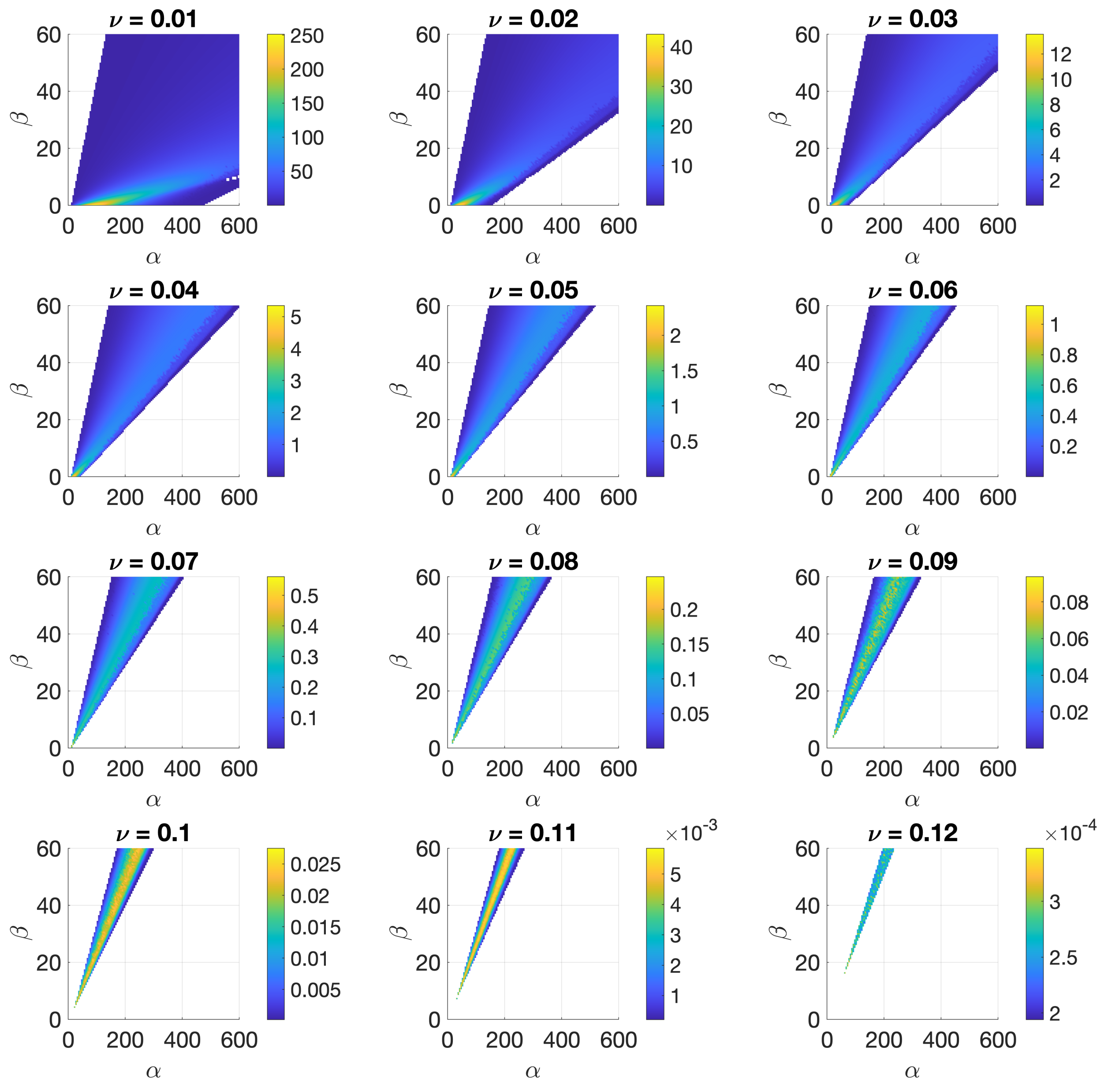

Figure S5: In case of bistability, the potential barriers $h_1 = h_2$ decrease with ν. Colors indicate the value of $h_1 = h_2$.

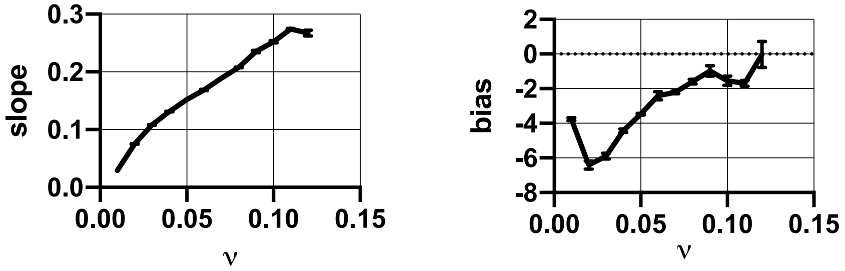

Figure S6: The potential barriers $h_1 = h_2$ are maximized in the middle region between the constraints $q_1(\nu) = 1$ and $q_2(\nu) = 1$ (Figure S5), and the relationship between α and β at these points is captured by a linear constraint with the displayed slope and bias. The linear fit was obtained for the data presented in Figure S5 using the built-in MATLAB function `fitlm`, yielding models with Pearson correlation coefficient $R^2 > 0.99$.

This reveals, for instance, that while increasing β in the bistable region eventually pushes the dynamics towards monostability, it can also be used to compensate for the adverse effects of ν and increase robustness to noise, illustrated in Figure S7. In particular, as greater promoter leakiness decreases robustness to noise in the bistable region (Figure S5), increasing β can be leveraged to mitigate this effect.

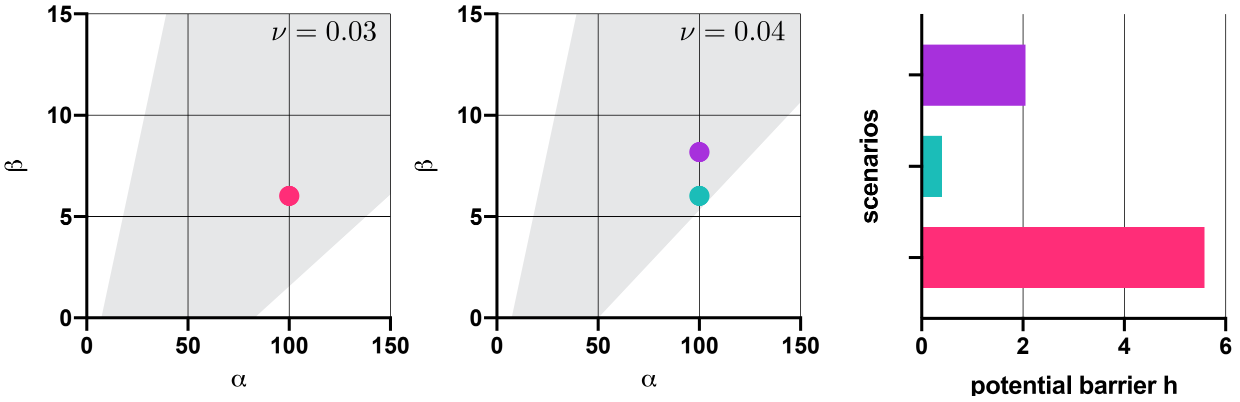

Figure S7: Increasing β can compensate for greater promoter leakiness. Simulation parameters are $\alpha = 100$, together with $\beta = 6$ and $\beta = 8$ for red/green and purple, respectively. Gray area denotes the region where the dynamics are bistable.

Finally, as illustrated in Figure S8, in the tristable region ($q_2(\nu) < 1$ and $q_3(\nu) > 1$ from Section S2.4) robustness of the middle stable fixed point is dominated by that of the other two near the region of bistability, and this relationship is reversed near the region of monostability.

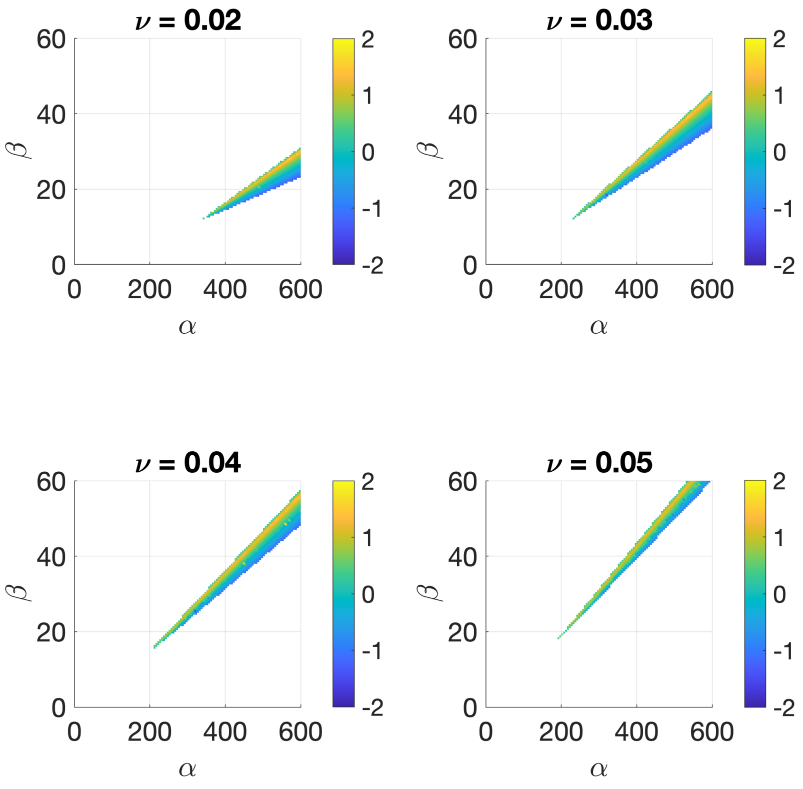

Figure S8: Relative height of the potential barriers $h_1 = h_2$ and h_3 in case of tristability. Colors indicate the value of $\log_{10}(h_3/h_1) = \log_{10}(h_3/h_2)$. The potential barriers $h_1 = h_2$ dominate h_3 near the region of bistability ($q_1(\nu) = 1$, top border) and vice versa near the region of monostability ($q_3(\nu) = 1$, bottom border).

S4 Population-level stability and robustness properties

Considering the results of the preceding two sections, the population-level stability and robustness properties of (S15) are governed by the random variables

$$Q = \frac{2(1+\beta)}{\alpha}, \qquad Q_i = \frac{\beta - b_i(\nu)}{\alpha a_i(\nu)}, \qquad i = 1, 2, 3, \tag{S21}$$

where $X = (\alpha, \beta) \sim \mathcal{N}(\mu, \Sigma)$ is considered to be a bivariate normal random variable with

$$\mu = \begin{pmatrix} \mu_\alpha \\ \mu_\beta \end{pmatrix}, \qquad \Sigma = \begin{bmatrix} \sigma_\alpha^2 & \rho\sigma_\alpha\sigma_\beta \\ \rho\sigma_\alpha\sigma_\beta & \sigma_\beta^2 \end{bmatrix}, \tag{S22}$$

as introduced in the manuscript. Therefore, in what follows we calculate the (approximate) distribution of random variables of the form

$$W = \frac{AX + B}{CX + D}, \tag{S23}$$

where X is a bivariate normal random variable with $A, C \in \mathbb{R}^2$ and $B, D \in \mathbb{R}$. Leveraging this, we next reveal how ν together with parameters in (S22) shape the population-level properties of (S15) in the presence of cell-to-cell heterogeneity [11].

S4.1 Distribution of the ratio of normal random variables

Although the distribution of W in (S23) could be approximated by that of a normal random variable [12, 13], this approximation is only accurate in a narrow parameter region. To obtain a more general approximation of the distribution of W revealing the role of the entries in μ and Σ, we next consider the result of Hinkley [14] and Geary [15] in a more general format, originally presented in [16]. In particular, let $X \sim \mathcal{N}(\mu, \Sigma)$ where $\mu \in \mathbb{R}^N$ and $\Sigma \in \mathbb{R}^{N\times N}$.

Introduce $A, C \in \mathbb{R}^N$ and $B, D \in \mathbb{R}$, together with

$$F(w) = \Phi\left(\frac{w - \mu_W(w)}{\sigma_W(w)}\right) \tag{S24}$$

where $\Phi(\cdot)$ is the cumulative density function of the standard normal distribution and

$$\mu_W = \frac{A\mu + B}{C\mu + D}, \qquad \sigma_W(w) = \sqrt{\frac{A - Cw}{C\mu + D}\Sigma\frac{A^\top - C^\top w}{C\mu + D}}.$$

With this, the cumulative density function of W from (S23) can be approximated [16] as

$$|\mathbf{P}(W < w) - F(w)| \leq \Phi\left(-\frac{C\mu + D}{\sqrt{C\Sigma C^\top}}\right). \tag{S25}$$

S4.2 Approximate distribution of Q and Q_i

Considering (S24) together with the notation of Section S4.1, in case of the random variable Q in (S21) we have $A = [0 \quad 2]$, $B = 2$, $C = [1 \quad 0]$, and $D = 0$, yielding

$$\mathbf{P}(Q < q) \approx F(q) = \Phi\left(\frac{q - \mu_Q}{\sigma_Q(q)}\right), \tag{S26}$$

$$\mu_Q = \frac{2(1 + \mu_\beta)}{\mu_\alpha}, \qquad \sigma_Q(q) = \frac{\sqrt{q^2\sigma_\alpha^2 + 4\sigma_\beta^2 - 4q\rho\sigma_\alpha\sigma_\beta}}{\mu_\alpha}. \tag{S27}$$

Similarly, for Q_i from (S21) we have that $A_i = [0 \quad 1]$, $B_i = -b_i(\nu)$, $C_i = [\alpha_i(\nu) \quad 0]$, and $D_i = 0$, resulting in

$$\mathbf{P}(Q_i < q_i) \approx F_i(q_i) = \Phi\left(\frac{q - \mu_i}{\sigma_i(q)}\right), \tag{S28}$$

$$\mu_i = \frac{\mu_\beta - b_i(\nu)}{\mu_\alpha a_i(\nu)}, \qquad \sigma_i(q_i) = \frac{\sqrt{q^2\left[a_i(\nu)\sigma_\alpha\right]^2 - 2\rho q_i\left[a_i(\nu)\sigma_\alpha\right]\sigma_\beta + \sigma_\beta^2}}{a_i(\nu)\mu_\alpha}. \tag{S29}$$

S4.3 Population-level composition

To characterize the population-level composition, let p_{mono}, p_{bi} and p_{tri} denote the fraction of the population with monostable, bistable, and tristable stability profile, respectively. Alternatively, these quantities measure the probability of a given realization lying in the corresponding stability region in Figure 3 of the manuscript. As earlier, we assume that the probability of α and β taking on negative values is negligible. Furthermore, to simplify the notation, we denote by σ_q and σ_i the value of $\sigma_q(1)$ and $\sigma_i(1)$, respectively.

The probability p_{mono} comprises two components: let p'_{mono} and p''_{mono} denote the probabilities of lying in the monostable regions on the upper left and lower right corners in in Figure 3 of the manuscript, respectively, so that the probability of monostability is given by $p_{mono} = p'_{mono} + p''_{mono}$. From Figure 3 it follows that $p'_{mono} = \mathbf{P}(Q_1 > 1)$, hence from (S28) we obtain that

$$p'_{mono} = \mathbf{P}(Q_1 > 1) \approx 1 - \Phi\left(\frac{1-\mu_1}{\sigma_1}\right),$$

and since $p'_{mono} + p_{bi} = \mathbf{P}(Q_2 > 1)$ from Figure 3 in the manuscript, we also have from (S28) that the fraction of the population that is bistable is given by

$$p_{bi} = \mathbf{P}(Q_2 > 1) - \mathbf{P}(Q_1 > 1) \approx \Phi\left(\frac{1-\mu_1}{\sigma_1}\right) - \Phi\left(\frac{1-\mu_2}{\sigma_2}\right). \tag{S30}$$

To compute p''_{mono}, let $\bar{\alpha}$ denote the value of α where the constraints $q_2(\nu) = q_3(\nu) = 1$ intersect (at the tip of the tristable region in Figure 3 of the manuscript), given by $\bar{\alpha}(\nu) = [b_2(\nu) - b3(\nu)]/[a_3(\nu) - a_2(\nu)]$. With this, we have that $p''_{mono} = \mathbf{P}(Q_2 < 1, \alpha < \bar{\alpha}(\nu)) + \mathbf{P}(Q_3 < 1, \alpha > \bar{\alpha}(\nu))$. Regarding the first term, we obtain

$$\begin{aligned}
\mathbf{P}(Q_2 < 1, \alpha < \bar{\alpha}(\nu)) &= \int_{-\infty}^{\bar{\alpha}(\nu)} \mathbf{P}(Q_2 < 1, \alpha = \alpha')\mathrm{d}\alpha' \\
&= \int_{-\infty}^{\bar{\alpha}(\nu)} \mathbf{P}(Q_2 < 1 | \alpha = \alpha')\mathbf{P}(\alpha = \alpha')\mathrm{d}\alpha' \\
&= \int_{-\infty}^{\bar{\alpha}(\nu)} \mathbf{P}(\beta < a_2(\nu)\alpha' + b_2(\nu) | \alpha = \alpha')\mathbf{P}(\alpha = \alpha')\mathrm{d}\alpha',
\end{aligned}$$

and from the marginal probability of multivariate normal random variables we further have

$$\mathbf{P}(\beta < a_2(\nu)\alpha' + b_2(\nu)|\alpha = \alpha') = \Phi\left(\frac{a_2(\nu)\alpha' + b_2(\nu) - \mu'}{\sigma'}\right)$$

with $\mu' = \mu_\beta + \rho\,(\alpha' - \mu_\alpha)\,\sigma_\beta/\sigma_\alpha$ and $\sigma' = \sqrt{1-\rho^2}\sigma_\beta$, and since $\mathbf{P}(\alpha = \alpha') = \phi(\frac{\alpha'-\mu_\alpha}{\sigma_\alpha})$, we obtain that

$$\mathbf{P}(Q_2 < 1, \alpha < \bar{\alpha}(\nu)) = \int_{-\infty}^{\bar{\alpha}(\nu)} \Phi\left(\frac{a_2(\nu)\alpha' + b_2(\nu) - \mu'}{\sigma'}\right)\phi\left(\frac{\alpha' - \mu_\alpha}{\sigma_\alpha}\right)\mathrm{d}\alpha',$$
$$\mathbf{P}(Q_3 < 1, \alpha > \bar{\alpha}(\nu)) = \int_{\bar{\alpha}(\nu)}^{\infty} \Phi\left(\frac{a_3(\nu)\alpha' + b_3(\nu) - \mu'}{\sigma'}\right)\phi\left(\frac{\alpha' - \mu_\alpha}{\sigma_\alpha}\right)\mathrm{d}\alpha',$$

eventually yielding

$$\begin{aligned} p_{mono} = &\int_{-\infty}^{\bar{\alpha}(\nu)} \Phi\left(\frac{a_2(\nu)\alpha' + b_2(\nu) - \mu'}{\sigma'}\right)\phi\left(\frac{\alpha' - \mu_\alpha}{\sigma_\alpha}\right)\mathrm{d}\alpha' + 1 \\ &+ \int_{\bar{\alpha}(\nu)}^{\infty} \Phi\left(\frac{a_3(\nu)\alpha' + b_3(\nu) - \mu'}{\sigma'}\right)\phi\left(\frac{\alpha' - \mu_\alpha}{\sigma_\alpha}\right)\mathrm{d}\alpha' - \Phi\left(\frac{1-\mu_1}{\sigma_1}\right). \end{aligned} \tag{S31}$$

Finally, since $1 = p_{mono} + p_{bi} + p_{tri}$ we obtain that that tristable fraction of the population is given by $p_{tri} = 1 - p_{mono} - p_{bi}$ where p_{mono} and p_{bi} are given in (S30) and (S31), respectively.

Considering that the bistable region shrinks as ν increases (Figure S4D), we expect p_{bi} to decrease with ν, confirmed in Figure 6A in the manuscript. Additionally, our results also reveal that while increasing μ_β when $\nu = 0$ always increases the probability of monostability (thus pushing the population towards unimodality), this is not necessarily true when $\nu > 0$. As illustrated in Figure 6B in the manuscript, with more details presented in Figure S9, while the unwanted middle peak (corresponding to $y \approx z$ at the steady state) can be eliminated by increasing μ_β in the green/purple cases in Figure S9, this choice is exactly the opposite of the required strategy in the red case in Figure S9.

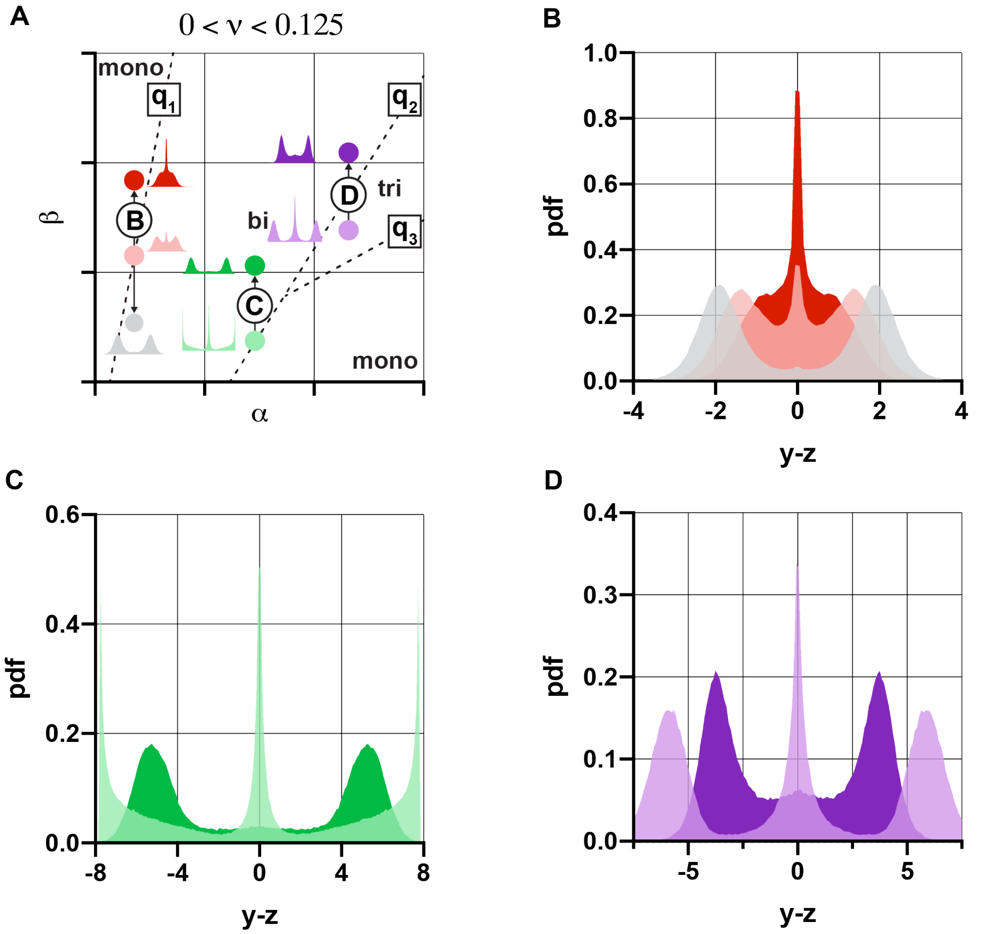

Figure S9: The composition of the population depends on the position of the pdf of (α, β) with respect to the regions uncovered in Figure 3 of the manuscript. (A) Increasing μ_β can shift the population away from bistability (red, illustrated in panel B) or towards it (green and purple, illustrated in panels C and D, respectively). (B) Increasing μ_β renders the population unimodal, whereas decreasing it gives rise to bimodality ($\mu_\alpha = 60$, light: $\mu_\beta = 21$, dark: $\mu_\beta = 24$, grey: $\mu_\beta = 18$). (C) Increasing μ_β renders the population bimodal ($\mu_\alpha = 60$, light: $\mu_\beta = 3.5$, dark: $\mu_\beta = 8$). (D) Increasing μ_β renders the population bimodal ($\mu_\alpha = 150$, light: $\mu_\beta = 20$, dark: $\mu_\beta = 30$). Simulation parameters: $\rho = 0.75$, $\sigma_\alpha = \mu_\alpha/5$, $\sigma_\beta = \mu_\beta/5$ in all panels.

S5 Context effects

Considering the rescaling $\alpha \leftarrow \alpha/(1+\beta_c)$ and $\beta \leftarrow \beta/(1+\beta_c)$, the dynamics in (S15) get transformed as if we had $\beta_c = 0$. Therefore, all results in the previous sections are readily applicable by simply applying the above rescaling.

For instance, in the absence of promoter leakiness ($\nu = 0$), the value of $q = 2(1+\beta)/\alpha$

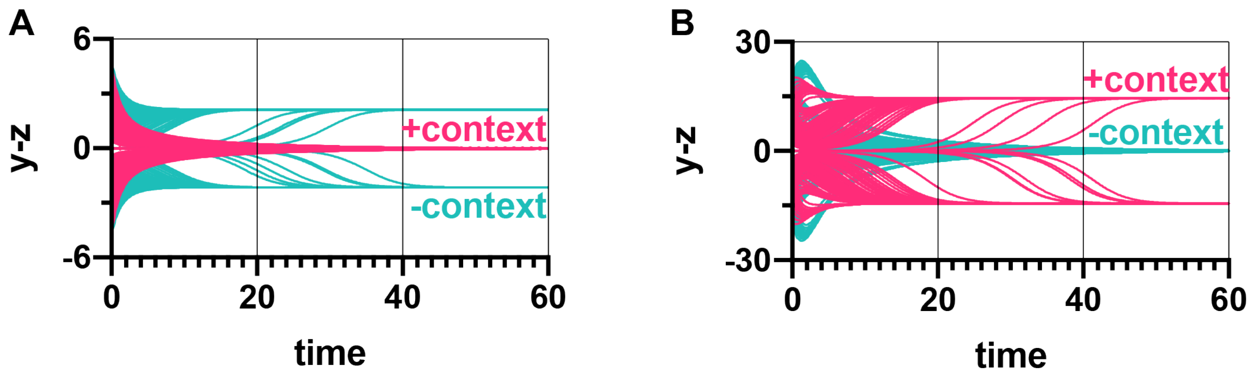

Figure S10: Loading from the context can cause transitions between stability profiles. (A) Without loading from the context (green, $\beta_c = 0$), the toggle switch lies in the bistable region ($\alpha = 50$, $\beta = 15$, $\nu = 0.03$), but once loading arises in the context (red, $\beta_c = 10$), the dynamics shift into the monostable region. (B) Without loading from the context (green, $\beta_c = 0$), the toggle switch lies in the monostable region ($\alpha = 150$, $\beta = 4$, $\nu = 0.03$), but once loading arises in the context (red, $\beta_c = 2$), the dynamics shift into the bistable region.

increases with β_c as $q = 2(1 + \frac{\beta}{1+\beta_c})(\frac{\alpha}{1+\beta_c})^{-1} = \frac{2(1+\beta)}{\alpha}(1 + \frac{\beta_c}{1+\beta})$. Additionally, the critical value of β_c pushing the dynamics from bistability to monostability (i.e., $q = 1$) is given by $\beta_c' = \frac{\alpha}{2} - (1 + \beta) = (1 + \beta)(q^{-1} - 1)$. From these two expressions it follows that greater values of β protect against unwanted effects of loading from the context [8] (as illustrated in Figure 7B in the manuscript). Similarly, in the presence of promoter leakiness ($\nu > 0$), loading from the context changes the value of $q_i(\nu)$ via the rescaling $\alpha \leftarrow \alpha/(1 + \beta_c)$ and $\beta \leftarrow \beta/(1+\beta_c)$, thus altering the stability profile of (S15). As a result, not only can bistable systems become monostable (Figure S10A), but the opposite is also possible (Figure S10B).

Population-level results presented in the previous section can be similarly extended for $\beta_c > 0$ by considering the values of Q and Q_i for $i = 1, 2, 3$ from (S21) via the rescaling $\alpha \leftarrow \alpha/(1+\beta_c)$ and $\beta \leftarrow \beta/(1+\beta_c)$, yielding the changes $\mu_\alpha \leftarrow \mu_\alpha/(1+\beta_c)$, $\mu_\beta \leftarrow \mu_\beta/(1+\beta_c)$, $\sigma_\alpha \leftarrow \sigma_\alpha/(1+\beta_c)$, and $\sigma_\beta \leftarrow \sigma_\beta/(1+\beta_c)$. With the corresponding modified values of μ_Q, σ_Q, μ_i, and σ_i from (S27) and (S29) the results of Section S4.3 are applicable even in the presence of loading from the context, so that we can study the population-level composition of (S15). In addition to obtaining the results presented in Figure 7A of the manuscript (with additional details provided in Figure S11), we can also reveal that simply by increasing loading from the

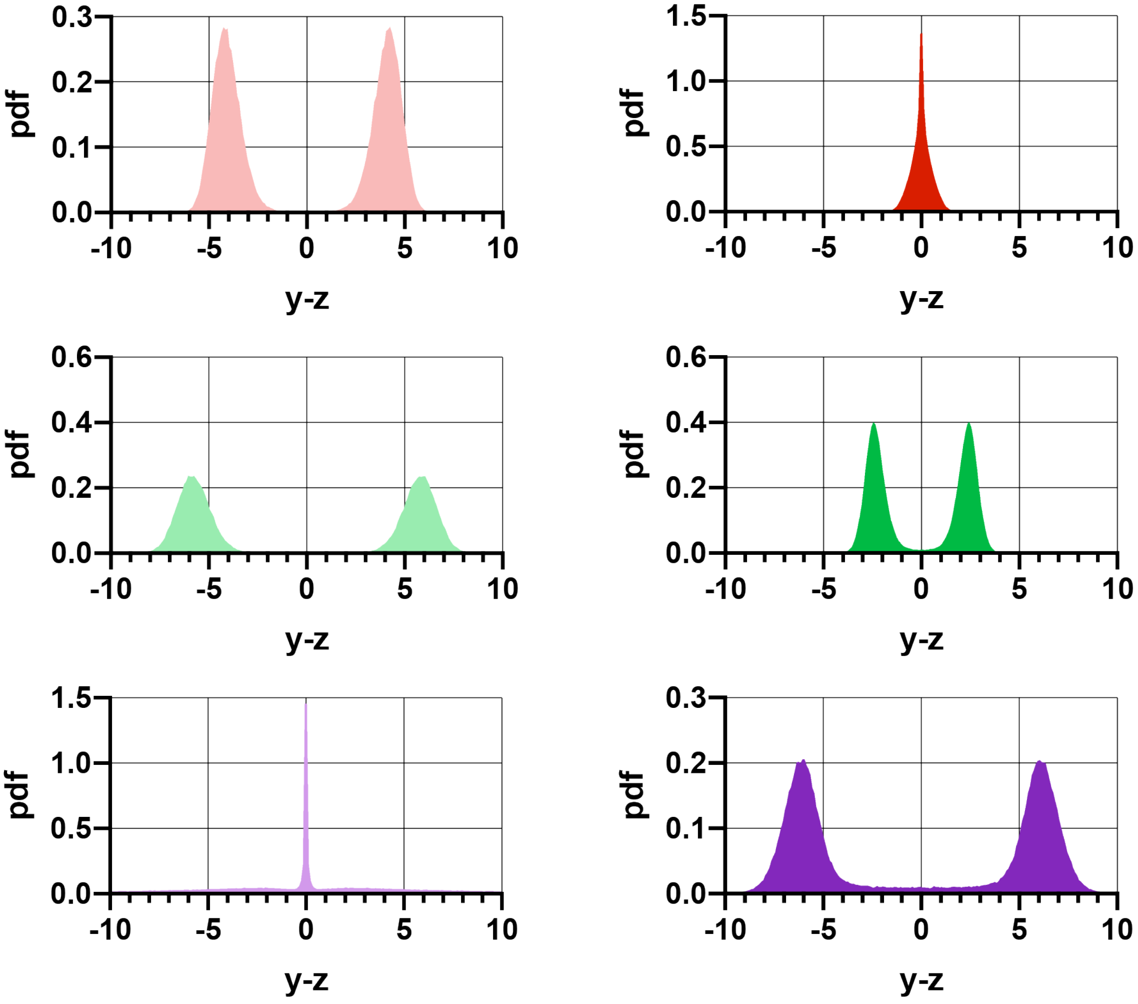

Figure S11: Detailed simulation data corresponding to Figure 7A in the manuscript. The histograms represent the distribution of $y - z$ in the steady state, obtained by generating 10,000 random samples with the following parameters: $\nu = 0.03$, $\sigma_\alpha = \mu_\alpha/5$, $\sigma_\beta = \mu_\beta/5$, $\sigma_c = \mu_c/5$, $\rho = \rho_1 = \rho_2 = 0.75$, and $(\mu_\alpha, \mu_\beta, \mu_c) = (10, 1, 0)$ for light red, $(\mu_\alpha, \mu_\beta, \mu_c) = (10, 1, 3)$ for dark red, $(\mu_\alpha, \mu_\beta, \mu_c) = (20, 2, 0)$ for light green, $(\mu_\alpha, \mu_\beta, \mu_c) = (20, 2, 3)$ for dark green, $(\mu_\alpha, \mu_\beta, \mu_c) = (150, 5, 0)$ for light purple, $(\mu_\alpha, \mu_\beta, \mu_c) = (150, 5, 15)$ for dark purple.

the context the dynamics can go through a sequence of unimodal-trimodal-bimodal-unimodal transitions (Figure S12).

Finally, the above results can be generalized to the case when β_c is also a random variable. In particular, let α, β, and β_c be distributed according to the multivariate normal distribution

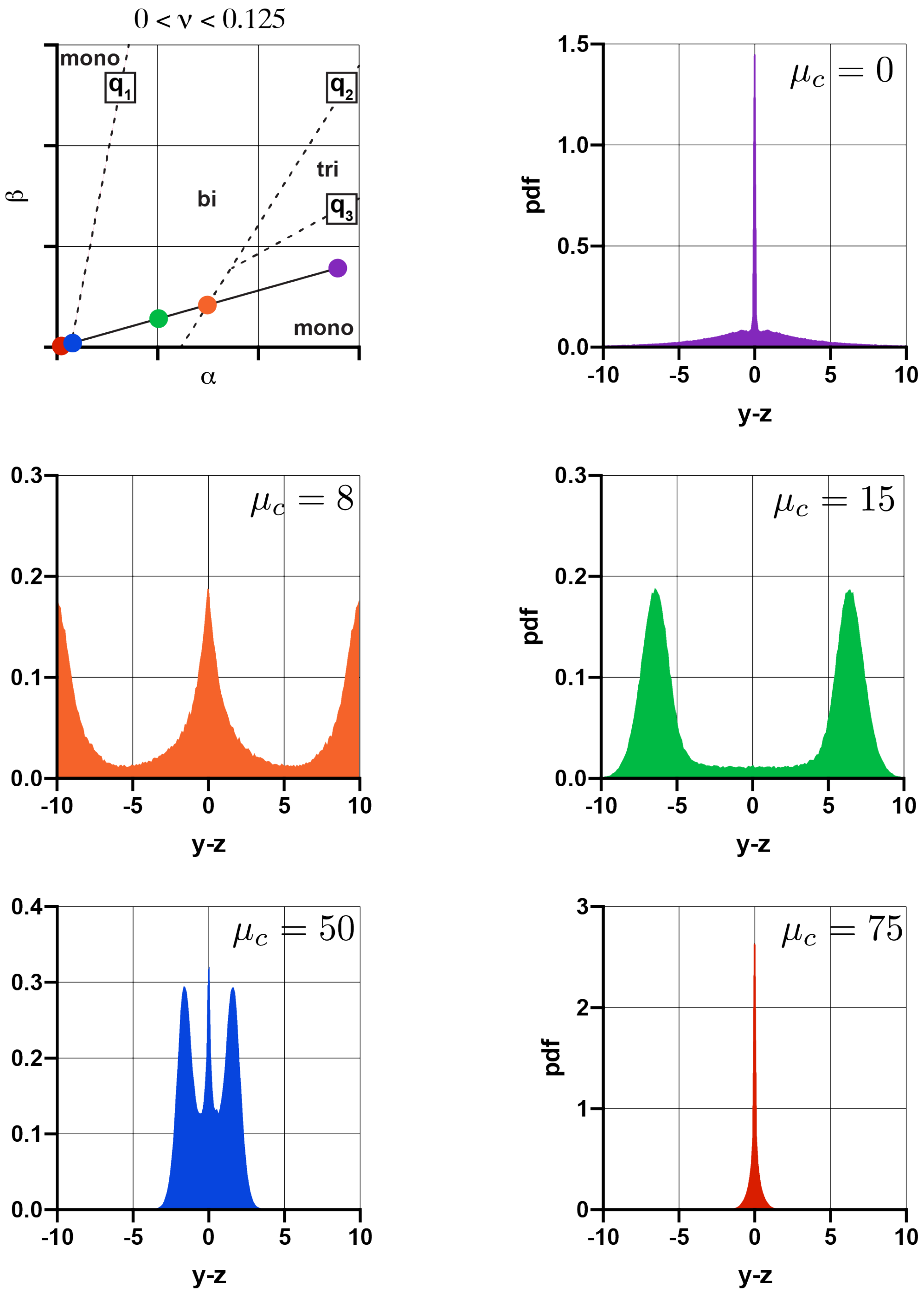

Figure S12: Loading from the context can give rise to trimodal stability profiles even in the absence of underlying tristable dynamics as a result of mixing bistable and monostable subpopulations. Simulation parameters are: $\nu = 0.03$, $\mu_\alpha = 150$, $\mu_\beta = 4$, $\rho = \rho_1 = \rho_2 = 0.75$, $\sigma_\alpha = \mu_\alpha/5$, $\sigma_\beta = \mu_\beta/5$, $\sigma_c = \mu_c/5$.

with mean and covariance

$$\mu = \begin{pmatrix} \mu_\alpha \\ \mu_\beta \\ \mu_c \end{pmatrix}, \qquad \Sigma = \begin{bmatrix} \sigma_\alpha^2 & \rho\sigma_\alpha\sigma_\beta & \rho_1\sigma_\alpha\sigma_c \\ \rho\sigma_\alpha\sigma_\beta & \sigma_\beta^2 & \rho_2\sigma_\beta\sigma_c \\ \rho_1\sigma_\alpha\sigma_c & \rho_2\sigma_\beta\sigma_c & \sigma_c^2 \end{bmatrix}. \tag{S32}$$

When $\nu = 0$, with the rescaling $\alpha \leftarrow \alpha/(1+\beta_c)$ and $\beta \leftarrow \beta/(1+\beta_c)$ we obtain that Q from (S21) takes the form

$$Q = \frac{2\left(1 + \frac{\beta}{1+\beta_c}\right)}{\alpha} = \frac{2(1+\beta+\beta_c)}{\alpha},$$

hence with the notation of Section S4.1 we have $A = [0 \quad 2 \quad 2]$, $B = 2$, $C = [1 \quad 0 \quad 0]$, and $D = 0$, so that from Section S4.2 it follows that (S26) still holds, but now with

$$\mu_Q = \frac{2(1+\mu_\beta+\mu_c)}{\mu_\alpha}, \quad \sigma_Q(q) = \frac{\sqrt{q^2\sigma_\alpha^2 - 4q\rho\sigma_\alpha\sigma_\beta - 4q\rho_1\sigma_\alpha\sigma_c + 4\sigma_\beta^2 + 8\rho_2\sigma_\beta\sigma_c + 4\sigma_c^2}}{\mu_\alpha}.$$

Similarly, when $\nu > 0$, the population-level stability and robustness properties of (S15) are governed by Q_i for $i = 1, 2, 3$ from (S21), which together with $\alpha \leftarrow \alpha/(1+\beta_c)$ and $\beta \leftarrow \beta/(1+\beta_c)$ yield

$$Q_i = \frac{\beta - (1+\beta_c)b_i(\nu)}{\alpha a_i(\nu)}.$$

Therefore, with the notation of Section S4.1 we now have $A = [0 \quad 1 \quad -b_i(\nu)]$, $B = -b_i(\nu)$, $C = [a_i(\nu) \quad 0 \quad 0]$, and $D = 0$, so that from Section S4.2 it follows that (S28) still holds, but now with $\mu_i = [\mu_\beta - b_i(1+\mu_c)]/(a_i\mu_\alpha)$ and

$$\sigma_i(q_i) = \frac{\sqrt{a_i q_i(a_i q_i \sigma_\alpha^2 - 2\rho\sigma_\alpha\sigma_\beta + b_i\rho_1\sigma_\alpha\sigma_c) + b_i(b_i\sigma_c^2 - 2\rho_2\sigma_\beta\sigma_c + a_i q_i \rho_1\sigma_\alpha\sigma_c) + \sigma_\beta^2}}{a_i\mu_\alpha}.$$

S6 Simulation parameters

Figure 1 Genetic context was considered by the addition of the repressilator [3], for details, see Section S1. Simulation parameters are $\alpha_1 = \alpha_2 = \alpha_3 = 10$, $\beta_1 = \beta_2 = \beta_3 = 1$, $n = 4$, $\nu = 0$, together with with $(\alpha, \beta) = (4, 0.1)$ and $(\alpha, \beta) = (8, 1.2)$ for toggle #1 and toggle #2, respectively. Stochastic simulations were performed by considering the overdamped Langevin dynamics widely used in biomolecular simulations [17, 18]. Accordingly, the dynamics of the toggle switch are extended as

$$\dot{y} = f_y(y, z) + \sigma\xi_y, \qquad \dot{z} = f(z, y) + \sigma\xi_z, \tag{S33}$$

where σ regulates the intensity of the zero-mean δ-correlated Gaussian white noise (ξ_y, ξ_z), causing trajectories to leave the metastable fixed points. Stochastic simulations were carried out using an Euler-Maruyama scheme [19] with time step $\Delta t = 0.1$, simulation time horizon $T = 1,000$ and noise intensity $\sigma = 0.1$.

Figure 2 Only analytical results are presented, no simulation is included.

Figure 3 Sample trajectories of (S15) were generated with random initial conditions $(y_0, z_0) \in [0, \alpha]^2$. Simulation parameters corresponding to red, green, and purple are $(\alpha, \beta, \nu) = (200, 5, 0.03)$, $(\alpha, \beta, \nu) = (200, 15, 0.03)$, and $(\alpha, \beta, \nu) = (200, 9.8, 0.03)$, respectively.

Figure 4 The potential surface in Figure 4A corresponds to $(\alpha, \beta) = (20, 0)$, computed using the algorithm outlined in Section 2.3 of the manuscript. In Figure 4C the value of β increases from $\beta = 0$ to $\beta = 2$ while $\nu = 0$ is kept constant, whereas $\alpha = 5$, $\alpha = 7.5$, and $\alpha = 10$ for purple, green, and red, respectively. The displayed values are normalized to the maximal potential barrier ($\alpha = 10$, $\beta = 0$). In both panels of Figure 4D $\nu = 0.03$. In the left panel red, green, and purple correspond to $\alpha = 25$, $\alpha = 50$, and $\alpha = 75$, respectively. In the right panel the same values of α correspond to the different shades of red, green, and

purple. For all colors, light, medium, and dark shades correspond to noise power $\sigma = 1.0$, $\sigma = 1.1$, and $\sigma = 1.2$, respectively, averaging three independent simulations considering (S33). Stochastic simulations were carried out as in Figure 1, using an Euler-Maruyama scheme [19] with time step $\Delta t = 0.1$ and simulation time horizon $T = 100,000$.

Figure 5 Shaded regions denote distributions obtained by considering 10,000 random samples of $Q = 2(1+\beta)/\alpha$ from (S22), whereas lines depict the corresponding approximations using (S26). In Figure 6A $\mu_\alpha = 10$, $\sigma_\alpha = \mu_\alpha/5$, $\sigma_\beta = \mu_\beta/5$, $\rho = 0$. In Figure 6B $\mu_\alpha = 10$, $\sigma_\alpha = \mu_\alpha/5$, $\mu_\beta = 2$, and $\sigma_\beta = \mu_\beta/5$, together with $\rho = -1$ for red, $\rho = -1/3$ for green, $\rho = 1/3$ for purple, and $\rho = 1$ for orange.

Figure 6 In Figure 6A, for each value of ν, first 100 triplets of $(\mu_\alpha, \mu_\beta, \rho) \in [0, 100] \times [0, 50] \times [-1, 1]$ are chosen using a uniform distribution. With $\sigma_\alpha = \mu_\alpha/3$ and $\sigma_\beta = \mu_\beta/3$ the bistable fraction is computed using (S30). In Figure 6B, 100,000 random samples are generated according to (S22) such that $\nu = 0.05$, $\rho = 0.75$, $\sigma_\alpha = \mu_\alpha/5$, $\sigma_\beta = \mu_\beta/5$, together with the following choices of (μ_α, μ_β): $(60, 21)$ for light red, $(60, 24)$ for dark red, $(60, 18)$ for grey, $(60, 3.5)$ for light green, $(60, 8)$ for dark green, $(150, 20)$ for light purple, and $(150, 30)$ for dark purple. In Figure 6C, the simulation parameters are $\nu = 0.05$, $\mu_\alpha = 75$, $\sigma_\alpha = \mu_\alpha/5$, $\sigma_\beta = \mu_\beta/5$, $\rho = 0.75$, such that 100,000 samples of (α, β) were generated for each value of $\mu_\beta \in \{1, 2, \ldots, 40\}$ using (S22), whereas the colored dots correspond to the sample values $\mu_\beta \in \{5, 15, 25, 35\}$ for green, red, purple, and orange, respectively. In the middle panel, the bistable fraction of these samples were determined by considering the percentage that lies in the grey region in the left panel between the $q_1(\nu) = 1$ and $q_2(\nu) = 1$ constraints. In the right panel, the distribution of the potential barrier is computed using the algorithm detailed in Section 2.3 of the manuscript, and its median value is displayed (normalized to the maximal value).

Figure 7 In Figure 7A, light dots denote the location of (μ_α, μ_β) in the absence of the context, whereas the corresponding dark dots illustrate the shift due to the context. The histograms represent the distribution of $y - z$ in the steady state, obtained by generating 10,000 random samples considering (S32) such that $\nu = 0.03$, $\sigma_\alpha = \mu_\alpha/5$, $\sigma_\beta = \mu_\beta/5$, $\sigma_c = \mu_c/5$, $\rho = \rho_1 = \rho_2 = 0.75$, together with the following choices of $(\mu_\alpha, \mu_\beta, \mu_c)$: $(10, 1, 0)$ for light red, $(10, 1, 3)$ for dark red, $(20, 2, 0)$ for light green, $(20, 2, 3)$ for dark green, $(150, 5, 0)$ for light purple, $(150, 5, 15)$ for dark purple. In Figure 7B, the data is obtained considering both directly computing the potential surface using the algorithm outlined in Section 2.3 of the manuscript (circles), as well as the approximation $h \approx \psi_1(q^{-1} - 1)^{\psi_2}$ of the potential barrier with $(\psi_1, \psi_2) = (0.545, 2.039)$ (solid lines). Simulation parameters are: $(\alpha, \beta) = (10, 2)$ for green, $(\alpha, \beta) = (6.67, 1)$ for red, and $(\alpha, \beta) = (4, 0.2)$ for purple, respectively, together with $\nu = 0$ in all cases.

References

(1) Gyorgy, A.; Jiménez, J. I.; Yazbek, J.; Huang, H.-H.; Chung, H.; Weiss, R.; Del Vecchio, D. Isocost Lines Describe the Cellular Economy of Genetic Circuits. *Biophys J* **2015**, *109*, 639–646.

(2) Gardner, T. S.; Cantor, C. R.; Collins, J. J. Construction of a genetic toggle switch in E. coli. *Nature* **2000**, *403*, 339–42.

(3) Elowitz, M. B.; Leibler, S. A synthetic oscillatory network of transcriptional regulators. *Nature* **2000**, *403*, 335–338.

(4) Vecchio, D. D.; Murray, R. M. *Biomolecular Feedback Systems*; Princeton University Press, 2014.

(5) Gyorgy, A.; Murray, R. M. Quantifying resource competition and its effects in the TX-TL system. 55th IEEE Conference on Decision and Control (CDC). 2016; pp 3363–3368.

(6) Gyorgy, A. Bistability requires better balanced toggle switches in the presence of competition for shared cellular resources. American Control Conference (ACC). 2019; pp 154–1546.

(7) Yong, C.; Gyorgy, A. Stability and Robustness of Unbalanced Genetic Toggle Switches in the Presence of Scarce Resources. *Life* **2021**, *11*.

(8) Gyorgy, A. Sharing Resources Can Lead to Monostability in a Network of Bistable Toggle Switches. *IEEE Control Systems Letters* **2018**, *3*, 308–313.

(9) Bhattacharya, S.; Zhang, Q.; Andersen, M. E. A deterministic map of Waddington's epigenetic landscape for cell fate specification. *BMC Systems Biology* **2011**, *5*, 85.

(10) Gyorgy, A. Scarcity of Cellular Resources Decreases the Robustness of Toggle Switches to Noise. American Control Conference (ACC). 2020.

(11) Elowitz, M. B.; Levine, A. J.; Siggia, E. D.; Swain, P. S. Stochastic Gene Expression in a Single Cell. *Science* **2002**, *297*, 1183–1186.

(12) Marsaglia, G. Ratios of Normal Variables and Ratios of Sums of Uniform Variables. *Journal of the American Statistical Association* **1965**, *60*, 193–204.

(13) Marsaglia, G. . Ratios of Normal Variables. *Journal of Statistical Software* **2006**,

(14) D.V. Hinkley, On the Ratio of Two Correlated Normal Random Variables. *Biometrika* **1969**, *56 (3)*, 635–639.

(15) Geary, R. C. *The Frequency Distribution of the Quotient of Two Normal Variates*; 1930; Vol. 93; pp 442–446.

(16) Gyorgy, A. How Cell-to-Cell Heterogeneity and Scarce Resources Shape the Population-Level Stability Profile of Toggle Switches. 58th IEEE Conference on Decision and Control (CDC). 2019; p k.

(17) Ando, T.; Meguro, T.; Yamato, I. Multiple Time Step Brownian Dynamics for Long Time Simulation of Biomolecules. *Molecular Simulation* **2003**, *29*, 471–478.

(18) Copperman, J.; Guenza, M. G. Coarse-Grained Langevin Equation for Protein Dynamics: Global Anisotropy and a Mode Approach to Local Complexity. *The Journal of Physical Chemistry B* **2015**, *119*, 9195–9211, PMID: 25356856.

(19) Higham., D. J. An Algorithmic Introduction to Numerical Simulation of Stochastic Differential Equations. *SIAM Review* **2001**, *43*, 525–546.
